# PINCH-1 promotes IGF-1 receptor expression and skin cancer progression through inhibition of the GRB10-NEDD4 complex

**DOI:** 10.7150/thno.70744

**Published:** 2022-02-28

**Authors:** Xiaoxiao Wang, Rong Wang, Kun Jiang, Maohua Zhu, Ling Guo, Chuanyue Wu

**Affiliations:** 1 Guangdong Provincial Key Laboratory of Cell Microenvironment and Disease Research, Shenzhen Key Laboratory of Cell Microenvironment, Department of Biology, and Academy for Advanced Interdisciplinary Studies, Southern University of Science and Technology, China; 2 Department of Pathology, University of Pittsburgh School of Medicine, Pittsburgh, PA 15261, USA

**Keywords:** PINCH-1, IGF-1 receptor, NEDD4, GRB10, Skin cancer

## Abstract

**Background:** Insulin-like growth factor 1 receptor (IGF-1R) expression and signaling play important roles in promotion of skin cancer progression. Identification of signaling pathways that regulate IGF-1R is crucial for understanding the pathogenesis and therapeutic treatment of skin cancer.

**Methods:** Molecular, cellular and genetic approaches were used to investigate the function of PINCH-1 in regulation of IGF-1R expression and skin cell behavior. Furthermore, conditional PINCH-1 knockout mouse and carcinogen (7, 12-dimethylbenz[a]anthracene (DMBA)/12-O-tetradecanoylphorbol-13-acetate (TPA))-induced skin cancer model were employed to determine the function of PINCH-1 in regulation of IGF-1R expression and skin carcinogenesis *in vivo*.

**Results:** Knockdown of PINCH-1 from HaCaT keratinocytes or A431 squamous carcinoma cells diminished IGF-1R levels, suppressed cell proliferation and increased apoptosis. Re-expression of PINCH-1 in PINCH-1 knockdown cells restored IGF-1R expression, cell proliferation and survival. Furthermore, depletion of NEDD4 effectively reversed PINCH-1 deficiency-induced down-regulation of IGF-1R expression, cell proliferation and survival. Conditional knockout of PINCH-1 from keratin 5 (K5) positive keratinocytes in mice, like depletion of PINCH-1 from keratinocytes in culture, reduced the IGF-1R level. Using a mouse model of DMBA/TPA-induced skin cancer, we show that the levels of both PINCH-1 and IGF-1R were significantly increased in response to treatment with the carcinogens. Genetic ablation of PINCH-1 from the epidermis markedly reduced the IGF-1R expression and cell proliferation despite stimulation with DMBA/TPA, resulting in resistance to chemical carcinogen-induced skin cancer initiation and progression.

**Conclusions:** Our results reveal a PINCH-1-NEDD4-IGF-1R signaling axis that is critical for promotion of skin tumorigenesis and suggest a new strategy for therapeutic control of skin cancer progression.

## Introduction

Skin cancers, which include both melanoma and non-melanoma skin malignancies, are the most common form of cancer in the world 1. Non-melanoma skin cancer can be further divided into multiple subtypes including basal cell carcinoma (BCC) and squamous cell carcinoma (SCC) 2-4. Of these two subtypes, SCC is more aggressive, possesses a significant risk of metastasis, and exhibits high morbidity and mortality 5-8. The development and progression of skin cancers involve multiple signaling pathways including that of insulin like growth factor 1 receptor (IGF-1R). IGF-1R signaling promotes cancer cell proliferation, survival, migration, angiogenesis, hypoxia response and metastasis, and contributes to resistance to anti-cancer therapies 9-12. There is evidence suggesting that IGF-1R was overexpressed in tumoral areas of squamous cell carcinoma 13. Furthermore, IGF-1R expression exhibits different patterns in different histological degrees of SCC: it is located primarily in the cell membrane of highly-differentiated SCC but it is located in the cytoplasm of moderately differentiated SCC and in the nucleus of poorly differentiated SCC tumor cells 14. The strong and differential expression of IGF-1R in SCC suggests that IGF-1R might contribute to the carcinogenesis and progression of SCC. Indeed, previous studies have demonstrated that increased expression of IGF-1 augments the susceptibility to chemical carcinogen-induced skin tumors, and suppression of IGF-1 signaling inhibits skin tumor formation 15, 16. Overexpression of IGF-1 in the keratin 5 (K5) positive epidermal basal cells activates IGF-1R signaling, which promotes downstream mitogenic and cell survival signaling, resulting in spontaneous skin tumor formation 15. Thus, IGF-1R may provide a useful therapeutic target for alleviation of SCC and other cancers 14, 17-23. Indeed, numerous efforts have been made to develop inhibitors that target IGF-1R signaling, some of which have exhibited a wide range of anti-tumor effects 18-20 and have entered clinical trials 21-23. One of the promising strategies for anti-IGF-1R therapy is to suppress its expression. Indeed, down-regulation of IGF-1R expression not only can suppress but also may reverse malignant transformation 24, 25. Thus, elucidation of the mechanisms that control IGF-1R expression is crucial for understanding the pathogenesis of skin tumors and may provide new strategies for prevention and treatment of these deadly diseases.

One of the mechanisms that control IGF-1R expression is through regulation of ubiquitination and consequently proteasome-mediated degradation of IGF-1R. Neural precursor cell-expressed developmentally down-regulated gene 4 (NEDD4) is one of the key E3 ubiquitin ligases that are involved in the ubiquitination and degradation of IGF-1R. NEDD4 contains an N-terminal C2 domain, three or four WW domains and a C-terminal HECT domain 26-28. NEDD4, through its N-terminal C2 domain, binds growth factor receptor bound protein 10 (GRB10) 29, 30. The interaction with NEDD4 is mediated by GRB10 SH2 domain, which simultaneously binds to the kinase domain of IGF-1R 31 and thereby promotes ubiquitination and degradation of IGF-1R 32-35. These findings suggest that NEDD4 works in concert with GRB10 and negatively regulates IGF-1R-dependent cell proliferation through stimulation of ubiquitination and internalization of IGF-1R 29, 36. Thus, the interaction between NEDD4 and GRB10 is critical for regulation of ubiquitination and degradation of IGF-1R and consequently IGF-1R expression and IGF-1R dependent signaling, cell proliferation and survival. How the interaction between NEDD4 and GRB10 is regulated, however, is incompletely understood.

PINCH-1 (particularly interesting new cysteine-histidine rich protein 1; also knowns as LIMS1 (Lims and senescent cell antigen-like domain 1)) is a widely expressed and evolutionally conserved cytoplasmic protein that functions in regulation of ECM adhesion, signaling, migration, proliferation and survival 37-56. The expression of PINCH-1 is elevated in many types of cancerous tissues, including that of breast, prostate, colon cancer, and lung 45, 57-59. At the molecular level, PINCH-1 interacts with several cytoskeletal and/or signaling proteins including integrin-linked kinase (ILK) 37, Nck-2 60, RSU-1 61, 62, EPLIN 50, myoferlin 53 and Notch-2 55. Interestingly, we recently found that PINCH-1 interacts with the C2 domain of Smurf1, a member of the NEDD4 family of E3 ubiquitin ligases 26 and regulates Smurf1-mediated degradation of bone morphogenetic protein receptor type II (BMPR2) 52. Given the structural similarity between the C2 domains of NEDD4 and Smurf1 and the functional importance of NEDD4 C2 domain in interaction with GRB10 and regulation of IGF-1R degradation, we sought to test whether PINCH-1 interacts with NEDD4 C2 domain and if so, its function in regulation of IGF-1R expression and IGF-1R-dependent processes including cell proliferation, survival and tumor growth. We report below our findings.

## Methods

### Animal studies

*PINCH-1^flox/flox^* transgenic mice were generated as previously described 54. *K5-Cre* mice were obtained from Shanghai Model Organisms Center, Inc. (MGI: 3050065). *K5-Cre* mice were crossed with the *PINCH-1^flox/flox^* mice to obtain *K5-Cre; Pinch1^flox/+^* mice. *K5-Cre; Pinch1^flox/+^* mice were then crossed with *PINCH-1^flox/flox^* mice to obtain *K5-Cre; Pinch1^flox/flox^* mice. For experimental purposes, male *K5-Cre; Pinch1^flox/flox^* mice were crossed with female *PINCH-1^flox/flox^* mice to obtain enough *K5-Cre; Pinch1^flox/flox^* mice (P1-K5) and *PINCH-1^flox/flox^* mice (control) (Figure 6A). The recombinant alleles were analyzed using genomic DNA extracted from the tips of mouse tails. Genotyping of *Cre* and floxed *PINCH-1* alleles was performed by PCR using oligonucleotide primers. PCR primers used for genotyping are listed in supplemental Table S1.

Tumors were induced by treatment of mice with DMBA and TPA as previously described protocol 63. Briefly, 25 mg DMBA (Sigma-Aldrich) in acetone was applied to a shaved area on mouse back skin. One week later, the same area of the mouse back skin was treated with 7.6 nmol TPA (Sigma-Aldrich) in 200 μL of acetone (twice a week for up to 24 weeks). All experiments were performed with the approval of the Institutional Animal Care and Use Committee, Southern University of Science and Technology.

### Cell culture, viral vector generation and infection

Human HaCaT keratinocytes and A431 squamous carcinoma cells were cultured in DMEM supplemented with 10% FBS (Gibco-Invitrogen), 50 U/mL penicillin and streptomycin at 37°C in 5% CO_2_. For generation of lentiviral vectors expressing short hairpin RNA (shRNA) targeting *PINCH-1*, the pLKO.1-TRC, psPAX2 and pMD2.G vectors were obtained from Addgene (plasmid #10878, plasmid #12260, and plasmid #12259). The pLKO.1-TRC vectors expressing shRNAs targeting human *PINCH-1* (Sh-P1 and Sh-P1') or control scrambled shRNA (Sh-con) were generated using the following sequences: Sh-P1, 5'- GAGGACCTATATGAATGG-3'; Sh-P1', 5'-AAGGTGATGTGGTCTCTGCTC-3'; Sh-con, 5'-ACGCATGCATGCTTGCTTT-3'. Lentiviruses encoding the above shRNAs were generated by co-transfection of HEK293T cells with pLKO.1 encoding the shRNAs, psPAX2 and pMD2.G vectors. To generate DNA expression vectors (i.e., pLVX-PINCH-1, pLVX-mCherry-GRB10, pLVX-3×FLAG-PINCH-1, pLVX-3×FLAG-NEDD4, pLVX-3×FLAG-NEDD4-ΔC2 and pLVX-3×FLAG-IGF-1R vectors), cDNAs encoding the corresponding protein sequences were cloned into the pLVX-IRES-Hyg, 3×FLAG tagged pLVX-IRES-Hyg (pLVX-3×FLAG) or pLVX-mCherry-C1 vectors. To generate lentiviral expression vectors encoding PINCH-1, mCherry-GRB10, 3×FLAG-PINCH-1, 3×FLAG-NEDD4, 3×FLAG-NEDD4-ΔC2 or 3×FLAG-IGF-1R were co-transfected with psPAX2 and pMD2.G into HEK293T cells. The sequence corresponding to the shRNA targeting region in the PINCH-1 expression vectors was changed to 5'-AAGGCGACGTCGTGTCTGCTC-3' to confer resistance to the Sh-P1. The sequences of all DNA inserts were verified by DNA sequencing (Invitrogen). For lentiviral infection, HaCaT and A431 cells were cultured in basal growth medium until 70% confluence and then replaced with fresh medium containing lentivirus at a multiplicity of infection (MOI) of 100 for 24 h. Lentiviral infections were carried out in the presence of 8 μg/mL polybrene.

### Western blotting (WB)

WB was performed as previously described 64, 65. For preparation of total protein lysates, cells or tissue (after grinding) were lysed in 1% SDS lysis buffer (25 mM Tris-HCl, pH6.8, 50mM DTT, 10% glycerin, 2.5% sucrose). Equal amounts (10-60 μg/lane) of total cell or tissue proteins were separated on 10% polyacrylamide gel and transferred onto a nitrocellulose membrane. Membranes were blocked for 1 h at room temperature in Tris-buffered saline (50 mM Tris-HCl and 150 mM NaCl, pH 7.4) containing 0.1% Tween 20 and 5% non-fat powdered milk, followed by overnight incubation at 4°C with HRP-conjugated mouse anti-GAPDH (Santa Cruz, sc-365062), HRP-conjugated mouse anti-FLAG-M2 HRP (Sigma-Aldrich, A8592 HRP), HRP-conjugated mouse anti-alpha Tubulin (Proteintech, HRP-66031), rabbit anti-PINCH-1 (Proteintech, 20772-1-AP), rabbit anti-NEDD4 (Proteintech, 21698-1-AP), rabbit anti-IGF-1Rβ (Cell Signaling, 9750S), rabbit anti-GRB10 (Proteintech, 23591-1-AP), rabbit anti-cleaved Caspase-3 (Cell Signaling, 9664S), mouse anti-mCherry (Signalway Antibody, T602), or mouse anti-His (Tiangen, AB102-02) antibodies (Abs). After washing and incubation with appropriate HRP-conjugated secondary anti-rabbit or mouse IgG Abs (Jackson ImmunoResearch, #711-005-152 or #715-005-151), the blots were developed using an ECL kit (Bio-Rad) or the Ultra ECL Western Blotting Detection Reagent (4A Biotech, 4AW011) and then exposed using an automatic digital gel image analysis system (Tanon, 6100B). Quantification of densitometry was performed using Image J. The levels of IGF-1R, NEDD4, GRB10, cleaved Caspase-3, and PINCH-1 relative to GAPDH, or Tubulin were calculated by quantification of the data from at least three independent experiments.

### RNA interference

siRNAs directed against human NEDD4 were synthesized by Invitrogen. The sense sequences of the siRNAs were as follows: NEDD4 siRNA (Si-NEDD4), 5'-GGAGUUGAUUAGAUUACAATT-3'; Si-NEDD4', 5'-CAUGAAUCUAGAAGAACATT-3'; and control siRNA (Si-NC), 5'-ACGCATGCATGCTTGCTTT-3'. HaCaT or A431 cells (1 × 10^5^ cells/mL in each well of the 6-well plates) were transfected with 25 pmol siRNA and 2 μL Lipofectamine RNAiMAX Transfection Reagent (Life Technologies) in each well of the 6-well culture dishes following the manufacturer's protocol.

### In situ proximity ligation assay (PLA)

HaCaT or A431 cells cultured on glass coverslips were fixed with 4% paraformaldehyde (PFA). PLA was performed with Duolink PLA technology probes and reagents (Sigma-Aldrich) following the manufacturer's instructions. Briefly, the samples were firstly washed with PBS twice and blocked by incubation with the supplied blocking solution for 60 min at 37°C in a wetbox. The samples were then incubated with pairs of primary rabbit and mouse Abs as specified in each experiment (e.g., pairs of rabbit anti-NEDD4 (Proteintech, 21698-1-AP) and mouse anti-GRB10 (Santa Cruz, sc74509) Abs or pairs of mouse anti-PINCH-1 (BD Biosciences, 612711) and rabbit anti-NEDD4 (Proteintech, 21698-1-AP) Abs) at 4°C overnight. In control experiments, one of the primary Ab was substituted with an irrelevant IgG (e.g., rabbit anti-NEDD4 Ab and irrelevant mouse IgG). After incubation, the coverslips were washed with buffer A twice, followed by incubation with the PLA probes (anti-mouse MINUS and anti-rabbit PLUS) for 60 min at 37°C. After washing twice times with buffer A, the ligation step was performed with ligase diluted in the ligation buffer for 30 min at 37°C. The samples were washed twice with buffer A, then the samples were incubated with amplification solution at 37°C for 100 min. After washing twice with buffer B for 10 min and once with 0.01× buffer B for 1 min, the coverslips were mounted with Duolink in situ mounting medium containing DAPI (H-1200; Vectashield) and observed under an SP8 confocal fluorescence microscope. At least 40 cells were analyzed for each experiment. All experiments were repeated at least three times. Positive PLA puncta per cell area were quantified by Image J.

### Co-immunoprecipitation (co-IP)

Cells (as specified in each experiment) infected with lentivirus encoding 3×FLAG, 3×FLAG-NEDD4, mCherry or mCherry-GRB10 were harvested and homogenized in the Western and IP lysis buffer (Beyotime, P0013) supplemented with protease inhibitor cocktail (Bimake, B14001) at 4ºC for 30 min. Protein concentration was measured using a Pierce BCA Protein Assay kit (Thermo Fish Scientific, 23227). Pre-cleared cell lysates (2-3 mg proteins/sample) were incubated with 15 μl/mL anti-Flag® M2 affinity gel (A2220, Merck) or 20 μl/mL anti-mCherry beads (KTSM, KTSM1331) at 4°C for 4-6 h, followed by washing 3 times with the lysis buffer. All samples were boiled with 1% SDS lysis buffer and then analyzed by WB. For endogenous Co-IP experiments, cell lysates were harvested by IP lysis buffer (Beyotime, P0013) supplemented with protease inhibitor (Roche, 04693132001). After incubating with lysis buffer at 4°C for 30 mins, cell lysates were pre-cleared with 40 μL of protein A/G-Sepharose beads (Santa Cruz, sc-2003) for 60 mins. Immunobeads were prepared by incubation of 50 μL of protein A/G-Sepharose beads with GRB10 (6 μg) Ab (Santa Cruz, sc-74509) or control mouse IgG (6 μg) for 2 h at 4°C, followed by washing with lysis buffer 3 times. Equal amounts of pre-cleared cell lysates (8 mg) were incubated with the immunobeads at 4°C overnight and then washed 3 times with the lysis buffer. The samples were eluted from Sepharose beads by boiling with 1% SDS lysis buffer and analyzed by WB. Protein bands were scanned by densitometry and quantified using Image J.

### GST fusion protein pull-down assay

For preparation of GST fusion proteins containing truncated forms of NEDD4, the corresponding NEDD4 cDNA sequences were cloned into the pGEX-4T-1 vector. For generation of maltose binding protein (MBP)-His-tagged PINCH-1 protein (MBP-PINCH-1), the cDNA encoding full length PINCH-1 was cloned into the pET-32M-MBP vector. The pGEX-4T-1 constructs encoding truncated forms of NEDD4 or the pET-32M-MBP construct encoding MBP-PINCH-1 were transfected into *Escherichia coli* BL21 (E. coli BL21). GST or GST-tagged proteins and MBP-PINCH-1 were purified from the corresponding E. coli BL21 lysates with Glutathione-Sepharose 4B matrix (GE Healthcare, 17-0756-01) and amylose resin kit (New England Biolabs, E8021S), respectively, following the manufacturers' instructions. Purified GST, GST-tagged proteins and MBP-PINCH-1 were analyzed by SDS-PAGE to verify their sizes and purity. For pull-down experiments, GST or GST-fusion proteins bound to Glutathione-Sepharose beads were incubated with recombinant MBP-PINCH-1 protein at 4°C overnight, followed by washing 3 times with PBS containing 1% Triton X-100. Proteins pulled down by GST or GST-fusion proteins were analyzed by WB.

### Immunofluorescence

Cells (as specified in each experiment) were seeded on fibronectin-coated coverslips in 24 well plates (2 × 10^4^ cells/well) and cultured overnight. The cells were then fixed with 4% PFA, washed 3 times with PBS and immersed in 0.1% Triton X-100 in PBS for 10 min at room temperature. After washing 3 times with PBS, the cells were incubated with rabbit anti-IGF-1R (Abcam, ab182408), guinea pig anti-Keratin 5 (PROGEN, GP-CK5), or rabbit anti-Ki67 (Cell Signaling, 12202) Abs at 4°C overnight. At the end of incubation, the cells were washed 3 times with PBS and incubated with the corresponding secondary Abs (Alexa Flours, Invitrogen) at 1:500 dilution at room temperature for 1 h. The coverslips were then washed 3 times with PBS for 2 min and mounted with Duolink in situ mounting medium containing DAPI. Images were acquired at 21°C using an SP8 confocal fluorescence microscope (20x dry objective 0.7 NA, 40x dry objective 0.85 NA or 63× oil objective 1.4 NA; Leica) with Leica X version 1.1.0.12420 image software. Skin tissues embedded in OCT were sectioned in 10 µm thickness. The frozen sections were analyzed by immunofluorescence staining as described above. The numbers of Ki67 positive cells or the fluorescence intensities of IGF-1R from five microscopic fields in each section were analyzed and quantified using Image J.

### QRT-PCR analysis

cDNAs were extracted from HaCaT or A431 cells using SuperPrep Cell Lysis Kit (Toyobo Life Science) following the manufacturer's protocol. QRT-PCR was performed using the primers (as specified in supplemental Table S2) in a 20-µL reaction volume in SYBR Green I Master Mix (Roche) on an ABI StepOne plus QPCR System. GAPDH mRNA was used as an internal control, which was quantified in parallel with mRNAs of the target genes. Normalization and fold changes were calculated by the ΔΔCt method.

### Cell proliferation

Cell proliferation was assessed by cell counting. HaCaT or A431 cells were seeded on a six well plate (1 × 10^5^ cells/well) and cultured in basal growth medium for 3 d, cell numbers were counted using Count Star.

### Apoptosis

Apoptosis was analyzed with the Annexin V-FITC/PI Apoptosis Detection Kit (4A Biotech) following the manufacturer's instructions. Briefly, cells (as specified in each experiment) were washed twice with cold PBS, and then resuspend in the Annexin V Binding Buffer at a concentration of 1-5 × 10^6^ cells/mL. Cell suspension was transferred to a 1.5 mL test tube (100 µL/tube), mixed with 5 µL of FITC Annexin V and incubated at room temperature for 5 min in the dark. The cell suspension was then mixed with 10 µL of Propidium Iodide Solution and 400 µL PBS, and immediately analyzed by FACSCalibur flow cytometer (BD Biosciences).

### Statistical analysis

Statistical analyses were performed using the GraphPad Prism 7.0 software. Data distribution was assumed to be normal, but this was not formally tested. Student's unpaired t-test was used to compare two groups. Multiple comparison test adopted One-way ANOVA with Tukey's post-hoc test. A p value <0.05 was considered significant.

## Results

### PINCH-1 forms a complex with NEDD4 in cells

**Figure 1:**
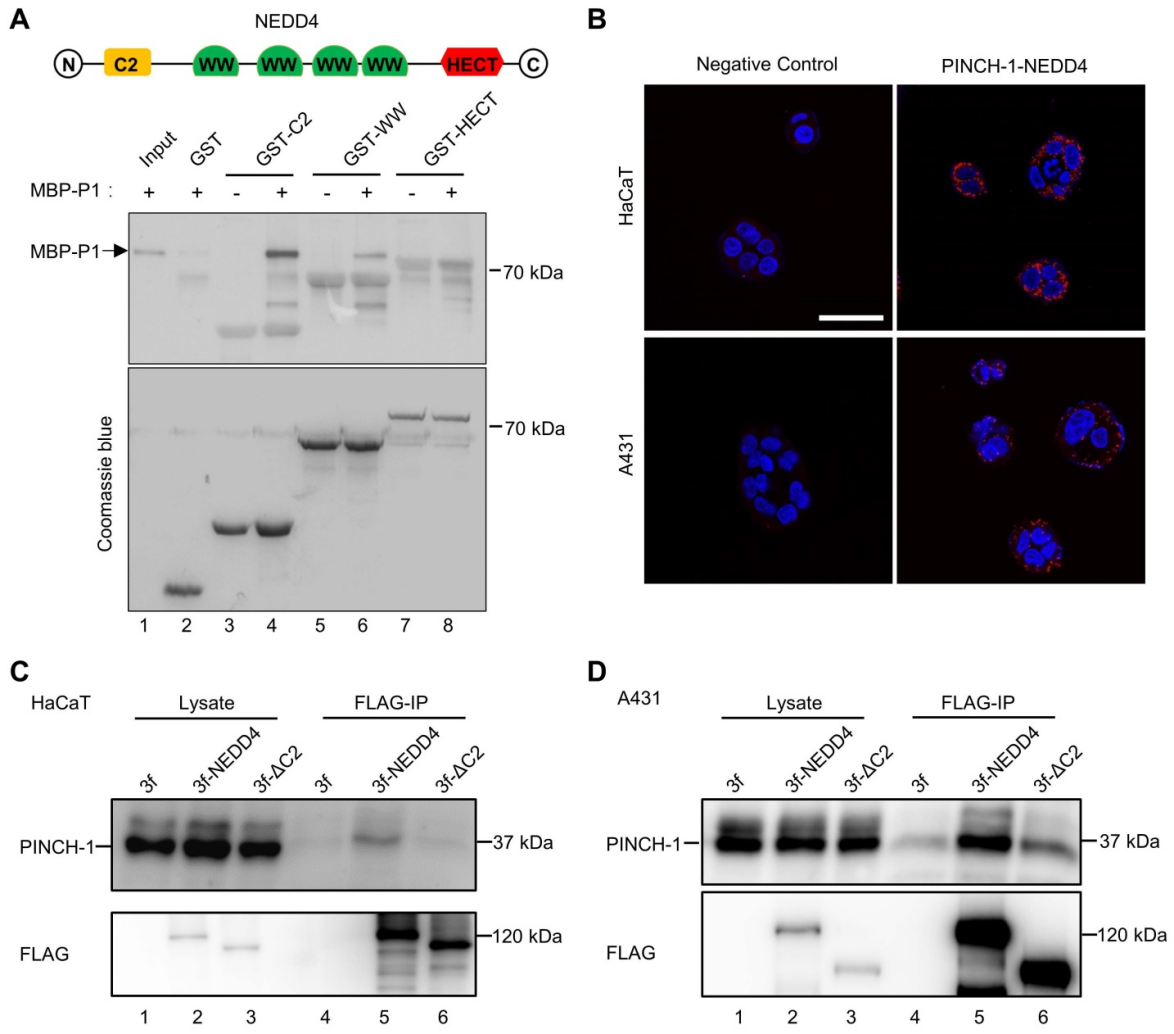
** PINCH-1 interacts with NEDD4. (A)** The interaction of MBP-PINCH-1 with GST-fusion protein containing different NEDD4 domains or GST alone as a negative control was analyzed by GST pull-down assay as described in the Methods. The MBP-PINCH-1 input (lane 1), GST (lane 2) or GST fusion protein pulldowns (lanes 3-8) as indicated in the figure were analyzed by WB with anti-His Ab (top panel). The control samples in lanes 3, 5 and 7 were prepared similarly to those in lanes 4, 6 and 8 except that MBP-PINCH-1(MBP-P1) were omitted. The same membrane was stained with coomassie blue to show the purity and positions of GST and GST-fusion proteins used in the pulldown assay. **(B)** PINCH-1 interaction with NEDD4 was analyzed by PLA in HaCaT (top panels) and A431 (bottom panels) cells. The nuclei were stained with DAPI. PLA signals (red) and DAPI staining (blue) were visualized under fluorescent microscopy. Scale bars = 50 μm. **(C-D)** Co-IP. HaCaT **(C)** and A431 **(D)** cells were infected with 3f, 3f-NEDD4 or 3f-ΔC2 lentiviral vectors, cultured in medium for 5 d, analyzed by co-IP with anti-FLAG Ab as described in the Methods. The cell lysates (lanes 1-3) and co-IP samples (lanes 4-6) were analyzed by WB with Abs recognizing FLAG or PINCH-1 as indicated in the figure.

To test whether PINCH-1 interacts with the C2 domain of NEDD4, we generated MBP-PINCH-1 and GST fusion proteins containing NEDD4 C2 (GST-C2), WW (GST-WW) or HECT (GST-HECT) domain. The interactions between MBP-PINCH-1 and GST-C2, GST-WW or GST-HECT were analyzed by GST-fusion protein pull down assay. The results showed that MBP-PINCH-1 was readily pulled down by GST-C2 (Figure 1A, lane 4) but not GST alone (Figure 1A, lane 2). A smaller amount of MBP-PINCH-1 was pulled down by GST-WW (Figure 1A, lane 6). By contrast, no MBP-PINCH-1 was pulled down by GST-HECT (Figure 1A, lane 8). These results suggest that NEDD4 C2 domain and to a less extent WW domain but not HECT domain interact with PINCH-1.

We next tested whether PINCH-1 and NEDD4 form a complex in cells. To do this, we performed PLA in HaCaT keratinocytes and A431 squamous carcinoma cells with anti-PINCH-1 and anti-NEDD4 Abs. Consistent with the results from the GST-fusion protein pulldown experiments, PINCH-1-NEDD4 complexes were detected in both HaCaT and A431 cells (Figure 1B, right panels). In control experiments, barely any PLA signals were detected in cells analyzed with irrelevant control IgGs (Figure 1B, left panels), confirming the specificity of the PLA assay. To confirm that PINCH-1 and NEDD4 form a complex in cells, we expressed 3×FLAG-tagged NEDD4 protein (3f-NEDD4), and 3×FLAG (3f) only as a control, in HaCaT and A431 cells and analyzed the interaction with PINCH-1 by co-IP. The results showed that PINCH-1 was readily co-immunoprecipitated with 3f-NEDD4 from HaCaT (Figure 1C, lane 5) and A431 cells (Figure. 1D, lane 5). No PINCH-1 was co-immunoprecipitated from the control cells infected with 3f lentivirus (Figure 1C-D, lane 4). Finally, consistent with the results of the GST-fusion protein pulldown experiments showing that NEDD4 C2 domain contains a major PINCH-1-binding site (Figure 1A), deletion of C2 from NEDD4 (3f-ΔC2) significantly reduced the ability of NEDD4 to interact with PINCH-1 (Figure 1C-D, compare lane 6 with lane 5). Collectively, these results suggest that PINCH-1 and NEDD4 form a complex in cells and the complex formation is mediated primarily by NEDD4 C2 domain, albeit NEDD4 WW domain may also contribute to the formation of this protein complex.

### PINCH-1 inhibits the interaction of NEDD4 with GRB10

**Figure 2:**
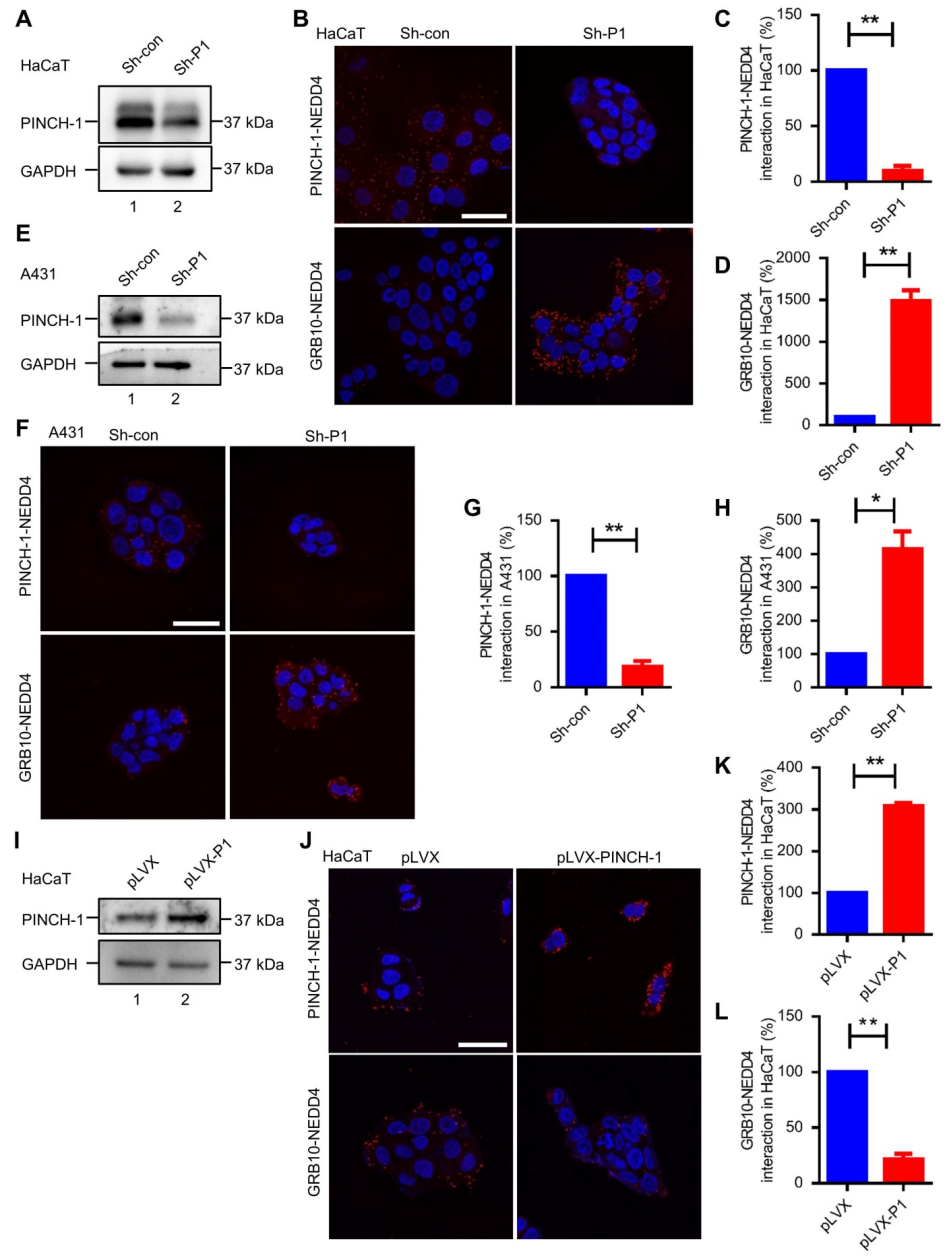
** PINCH-1 negatively regulates the interaction between NEDD4 and GRB10. (A-H)** HaCaT and A431 cells were infected with lentiviral vectors encoding Sh-P1 or Sh-con and cultured in medium for 5 d. The cells were analyzed by WB with Abs for PINCH-1 or GAPDH, or PLA with Abs for PINCH-1 and NEDD4 or GRB10 and NEDD4 as indicated in these figures. The lysates of the infected HaCaT **(A)** and A431 **(E)** cells were analyzed by WB. The PINCH-1-NEDD4 **(C)** and GRB10-NEDD4 **(D)** protein complexes in the infected HaCaT cells **(B)** were analyzed by PLA and quantified as described in the Methods. The PINCH-1-NEDD4 **(G)** and GRB10-NEDD4 **(H)** protein complexes in the infected A431 **(F)** cells were analyzed by PLA and quantified. **(I-L)** HaCaT cells were infected with lentiviral vectors encoding pLVX or pLVX-PINCH-1 (pLVX-P1) and cultured in medium for 5 d.** (I)** HaCaT cells were analyzed by WB with Abs for PINCH-1 and GAPDH as indicated in the figure. The PINCH-1-NEDD4 **(K)** and GRB10-NEDD4 **(L)** protein complexes in the infected HaCaT cells **(J)** were analyzed by PLA and quantified as described in the Methods. The PLA signals (red) and DAPI staining (blue) were visualized under fluorescent microscopy **(B, F and J)**. Scale = 50 μm. Data are presented as mean ± SEM using t-test analysis, *P < 0.05, **P < 0.01.

Previous studies have shown that NEDD4, through its C2 domain, interacts with IGF-1R binding protein GRB10 31, 66 and thereby promotes IGF-1R degradation 29, 36. Thus, our finding that PINCH-1 interacts with NEDD4 primarily through its C2 domain (Figure 1) raised an interesting possibility that PINCH-1 may regulate NEDD4 interaction with GRB10 and consequently IGF-1R degradation. To test this possibility, we knocked down PINCH-1 from HaCaT cells (Figure 2A, compare lane 2 with lane 1) and tested the effect on the GRB10-NEDD4 complex by PLA. As expected, knockdown of PINCH-1 significantly reduced the level of the PINCH-1-NEDD4 complex in HaCaT cells (Figure 2B, compare the top right panel with top left panel; Figure 2C). Importantly, the level of the GRB10-NEDD4 complex in HaCaT cells was markedly increased in response to depletion of PINCH-1 (Figure 2B, compare the bottom right panel with bottom left panel; Figure 2D). Similar results were obtained in A431 cells (Figure 2E-H). These results suggest that PINCH-1 negatively regulates the GRB10-NEDD4 complex formation.

**Figure 3:**
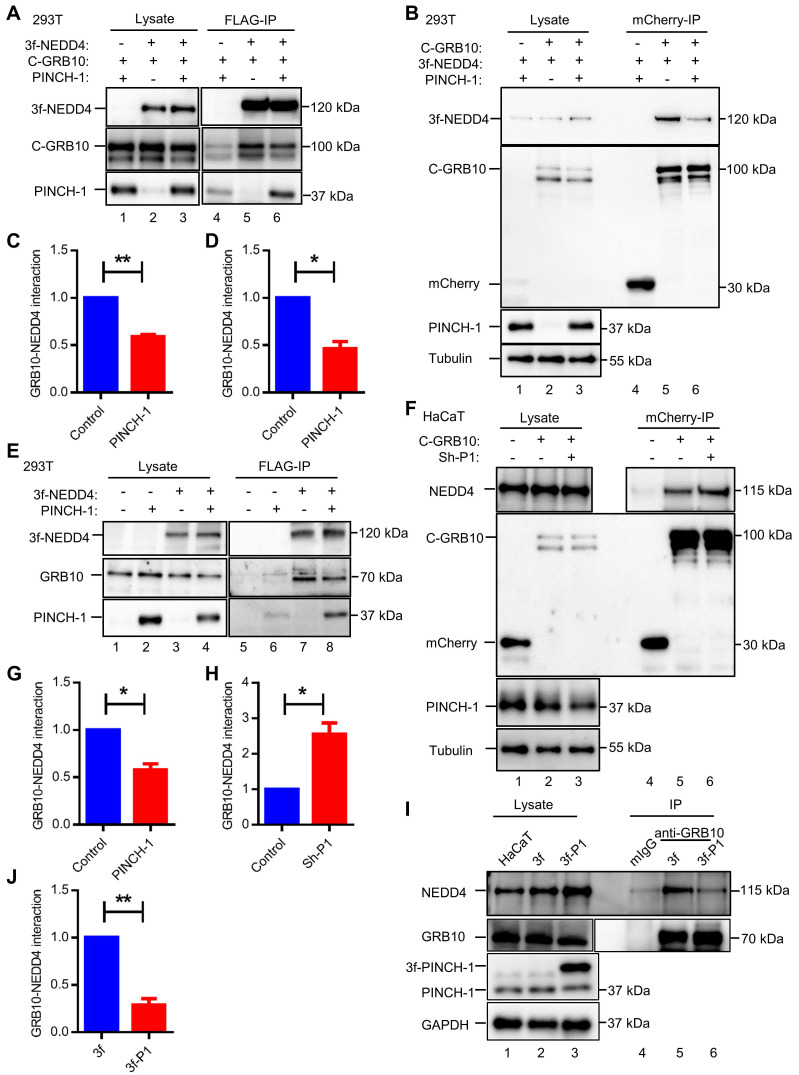
** Co-immunoprecipitation analyses of the GRB10-NEDD4 interaction. (A-D)** HEK293T cells were transfected with pLVX vectors encoding mCherry-GRB10 (C-GRB10), mCherry (as a control), 3f-NEDD4, 3f (as a control), and/or PINCH-1 as indicated in the figure for 24 h. The interaction between mCherry-GRB10 and 3f-NEDD4 was analyzed by IP with anti-FLAG **(A)** or anti-mCherry **(B)** Abs. The cell lysates (lanes 1-3) and the IP samples (lanes 4-6) were analyzed by WB with anti-FLAG, anti-mCherry, anti-PINCH-1 and anti-Tubulin Abs. The amount of mCherry-GRB10 co-immunoprecipitated with 3f-NEDD4 **(C)** or that of 3f-NEDD4 co-immunoprecipitated with mCherry-GRB10 **(D)** in the PINCH-1 overexpressing cells was quantified and compared to that in the control cells without PINCH-1 overexpression (Control) (normalized to 1, n = 3). **(E)** HEK293T cells were transfected with pLVX vectors encoding 3f-NEDD4, 3f (as a control), and/or PINCH-1 as indicated in the figure for 24 h. The interaction between GRB10 and 3f-NEDD4 was analyzed by IP with anti-FLAG Ab. The cell lysates (lanes 1-4) and the IP samples (lanes 5-8) were analyzed by WB with anti-FLAG, anti-GRB10 and anti-PINCH-1 Abs. **(G)** The amount of GRB10 co-immunoprecipitated with 3f-NEDD4 in the PINCH-1 overexpressing cells was quantified and compared with that in the control cells without PINCH-1 overexpression (normalized to 1, n = 3). **(F)** HaCaT cells were infected with mCherry-GRB10, mCherry (as a control) and Sh-P1 or Sh-con lentivirus and cultured in medium for 5 d. The interaction between NEDD4 and mCherry-GRB10 was analyzed by IP with mCherry Ab. The cell lysates (lanes 1-3) and the IP samples (lanes 4-6) were analyzed by WB with anti-FLAG, anti-mCherry, anti-PINCH-1 and anti-Tubulin Abs. **(H)** The amount of NEDD4 co-immunoprecipitated with mCherry-GRB10 in the PINCH-1 knockdown cells (Sh-P1) was quantified and compared to that in control Sh-con cells (normalized to 1, n = 3). **(I)** HaCaT cells were transfected with pLVX vectors encoding 3f-PINCH-1 (3f-P1) or 3f (as a control) lentiviral vectors and cultured in medium for 5 d. The interaction between endogenic GRB10 and NEDD4 was analyzed by co-IP with anti-GRB10 Ab or irrelevant mouse IgG (mIgG) (as a control). The cell lysates (lanes 1-3) and co-IP samples (lanes 4-6) were analyzed by WB with Abs recognizing NEDD4, GRB10, PINCH-1 or GAPDH as indicated in the figure. **(J)** The amount of NEDD4 co-immunoprecipitated with endogenic GRB10 in the 3f-P1 overexpressing cells was quantified and compared to that in the cells transfected with 3f lentiviral vectors (normalized to 1, n = 3). Data are presented as mean ± SEM using t-test analysis, *P < 0.05, **P < 0.01.

To further test this, we overexpressed PINCH-1 in HaCaT cells (Figure 2I, compare lane 2 with lane 1) and tested the effect on the GRB10-NEDD4 complex formation by PLA. The results showed that increased expression of PINCH-1 markedly increased the level of the PINCH-1-NEDD4 complex in HaCaT cells (Figure 2J, compare the top right panel with top left panel; Figure 2K). Importantly, the level of the GRB10-NEDD4 complex in these cells was significantly reduced (Figure 2J, compare the bottom right panel with bottom left panel; Figure 2K). Similar results were obtained with A431 cells (Figure S1A-D). Additionally, we overexpressed PINCH-1 in HEK293T cells expressing mCherry-tagged GRB10 and 3f-NEDD4 and analyzed the effects on the interaction between mCherry-tagged GRB10 and 3f-NEDD4 by co-IP with anti-FLAG or anti- mCherry Abs. Consistent with the PLA results (Figure 2I-L and Figure S1A-D), overexpression of PINCH-1 significantly reduced the amount of mCherry-tagged GRB10 that was co-immunoprecipitated with 3f-NEDD4 (Figure 3A, compare lane 6 with lane 5; Figure 3C). Similarly, the amount of 3f-NEDD4 that was co-immunoprecipitated with mCherry-tagged GRB10 was reduced in response to overexpression of PINCH-1 (Figure 3B, compare lane 6 with lane 5; Figure 3D). To confirm these results, we overexpressed PINCH-1 in HEK293T cell expressing 3f-NEDD4 and analyzed the amounts of endogenous GRB10 co-immunoprecipitated with 3f-NEDD4. Again, the amount of endogenous GRB10 co-immunoprecipitated with 3f-NEDD4 was reduced in response to increased expression of PINCH-1 (Figure 3E, compare lane 8 with lane 7; Figure 3G). Additionally, we knocked down PINCH-1 from HaCaT cells expressing mCherry-tagged GRB10 and found that the amount of endogenous NEDD4 co-immunoprecipitated with mCherry-tagged GRB10 was increased in response to knockdown of PINCH-1 (Figure 3F, compare lane 6 with lane 5; Figure 3H). Finally, we overexpressed 3f-PINCH-1 in HaCaT cells and analyzed the amounts of endogenous GRB10 co-immunoprecipitated with endogenous NEDD4. Again, the amount of endogenous NEDD4 co-immunoprecipitated with GRB10 was reduced in response to increased expression of 3f-PINCH-1 (Figure 3I, compare lane 6 with lane 5; Figure 3J). Collectively, these results suggest that PINCH-1 negatively regulates the interaction between NEDD4 and GRB10.

### PINCH-1 regulates IGF-1 receptor level in a NEDD4 dependent manner

**Figure 4:**
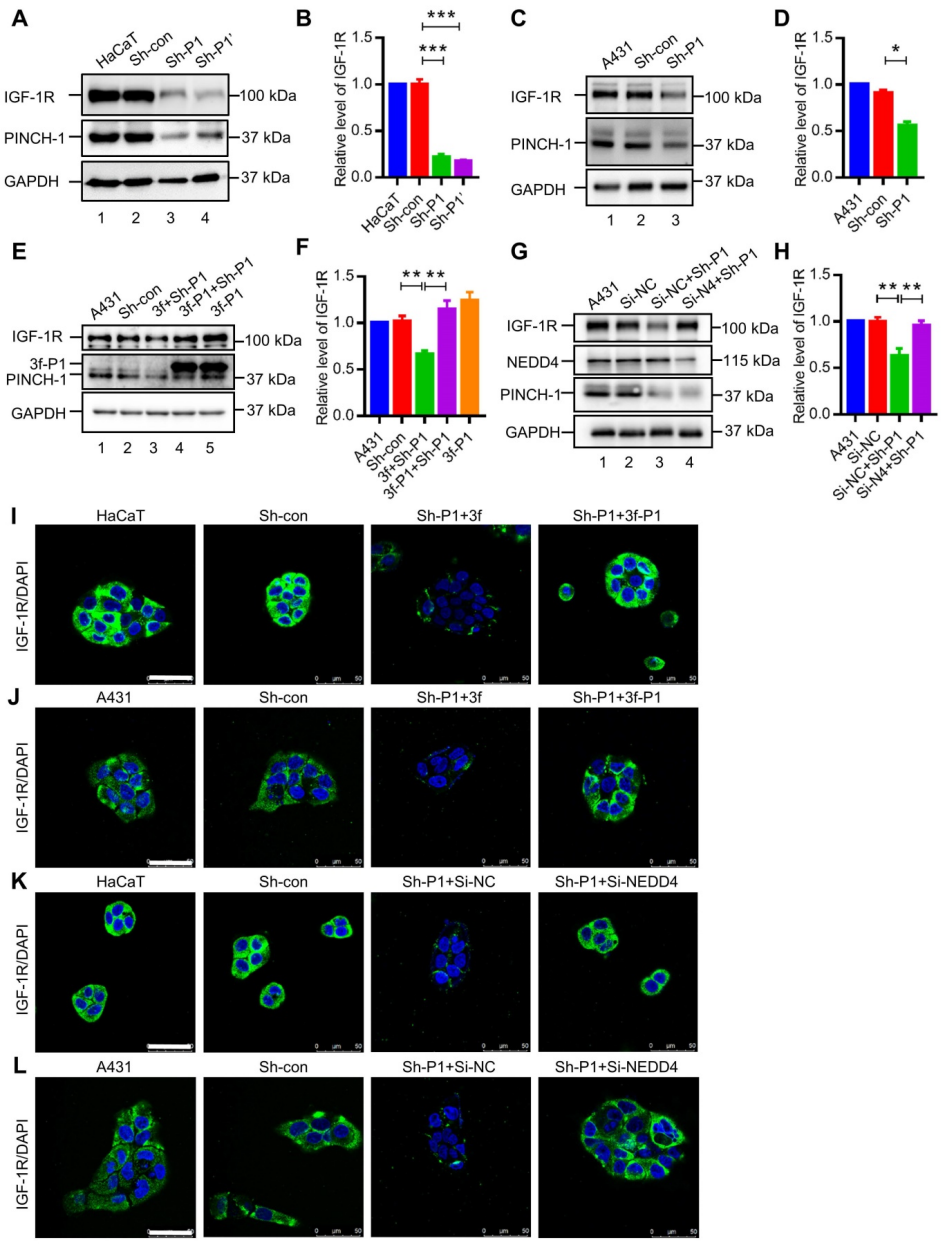
** PINCH-1-mediated regulation of IGF-1R expression is dependent on NEDD4. (A)** HaCaT cells were infected with Sh-P1, Sh-P1' or Sh-con lentivirus, cultured in medium for 5 d, and analyzed by WB with anti-IGF-1R, anti-PINCH-1 and anti-GAPDH Abs as indicated. **(B)** The IGF-1R levels in Sh-P1, Sh-P1' or Sh-con infected HaCaT cells were quantified and compared to those in the wild-type cells (normalized to 1, n = 3). **(C)** A431 cells were infected with Sh-P1 or Sh-con lentivirus, cultured in medium for 5 d, and analyzed by WB with Abs as indicated.** (D)** The IGF-1R levels in Sh-P1 or Sh-con infected A431 cells were quantified and compared to those in the wild-type cells (normalized to 1, n = 3). **(E-F)** A431 cells were infected with Sh-con or Sh-P1 for 2 d, and the Sh-P1 infectants were then infected with 3f or 3f-PINCH-1 (3f-P1) lentivirus. Three days later, the infectants and wild type A431 cells were analyzed by WB with Abs as indicated **(E)**. The IGF-1R levels in the infectants were quantified and compared to those in the wild-type cells (normalized to 1, n = 3) **(F)**.** (G-H)** A431 cells were infected with Sh-P1 or Sh-con lentivirus for 2 d. The Sh-P1 infectants were then transfected with Si-NEDD4 (Si-N4) or Si-NC for 2 d. The cells were analyzed by WB with Abs as indicated **(G)**. The IGF-1R levels in the PINCH-1 and/or NEDD4 knockdown cells were quantified and compared to those in the wild-type cells (normalized to 1, n = 3) **(H)**.** (I-J)** HaCaT** (I)** and A431 **(J)** cells were infected with Sh-con or Sh-P1 for 2 d, and the Sh-P1 infectants were then infected with 3f or 3f-P1 lentivirus as indicated in the figure. Three days later, the cells were immunofluorescently stained with DAPI (blue) and IGF-1R Ab (green) as described in the Methods. Scale bar = 50 μm. **(K-L)** HaCaT** (K)** and A431** (L)** cells were infected with Sh-P1 or Sh-con lentivirus for 2 d and transfected with Si-NEDD4 or Si-NC for 2 d as indicated in the figure. The cells were then immunofluorescently stained with DAPI (blue) and IGF-1R Ab (green). Scale bar = 50 μm. Data are presented as mean ± SEM using one-way ANOVA with Tukey's post-hoc analysis. *P < 0.05, **P < 0.01, ***P < 0.001.

Because the GRB10-NEDD4 complex is known to play an important role in regulation of the level of IGF-1R, the finding that PINCH-1 inhibits the GRB10-NEDD4 complex formation (Figure 2-3) prompted us to test whether PINCH-1 influences the level of IGF-1R. To do this, we knocked down PINCH-1 from HaCaT cells and analyzed the effect on the IGF-1R level by WB (Figure 4A-B) and immunofluorescence staining (Figure 4I). Consistent with an inhibitory effect of PINCH-1 on the GRB10-NEDD4 complex formation, depletion of PINCH-1 from HaCaT cells significantly reduced the protein (Figure 4A, compare lanes 3 and 4 with lane 2; Figure 4B) but not mRNA (Figure S2A) levels of IGF-1R. Similar results were obtained with A431 cells (Figure 4C, lane 3; Figure 4D; Figure S2B). Re-expressed 3f-PINCH-1 in PINCH-1 knockdown cells completely reversed the PINCH-1 deficiency-induced down-regulation of the IGF-1R level (Figure 4E, lane 4; Figure 4F), confirming the specificity of the knockdown experiments. Similar results were obtained with IGF-1R immunofluorescence staining of HaCaT (Figure 4I) and A431 (Figure 4J) cells. Thus, consistent with a negative role of PINCH-1 in regulation of NEDD4 interaction with GRB10, PINCH-1 positively regulates IGF-1R protein expression.

To test whether PINCH-1 mediated regulation of IGF-1R protein expression is dependent on NEDD4, we depleted NEDD4 from PINCH-1 knockdown A431 cells and analyzed the effect on IGF-1R protein expression. The results showed that depletion of NEDD4 completely restored the level of IGF-1R (Figure 4G, lane 4; Figure 4H). Similar results were obtained with IGF-1R immunofluorescence staining of HaCaT (Figure 4K) and A431 (Figure 4L) cells. These results suggest that PINCH-1 regulates IGF-1R protein expression through NEDD4.

### Depletion of NEDD4 reverses PINCH-1 deficiency-induced inhibition of cell proliferation and survival

**Figure 5:**
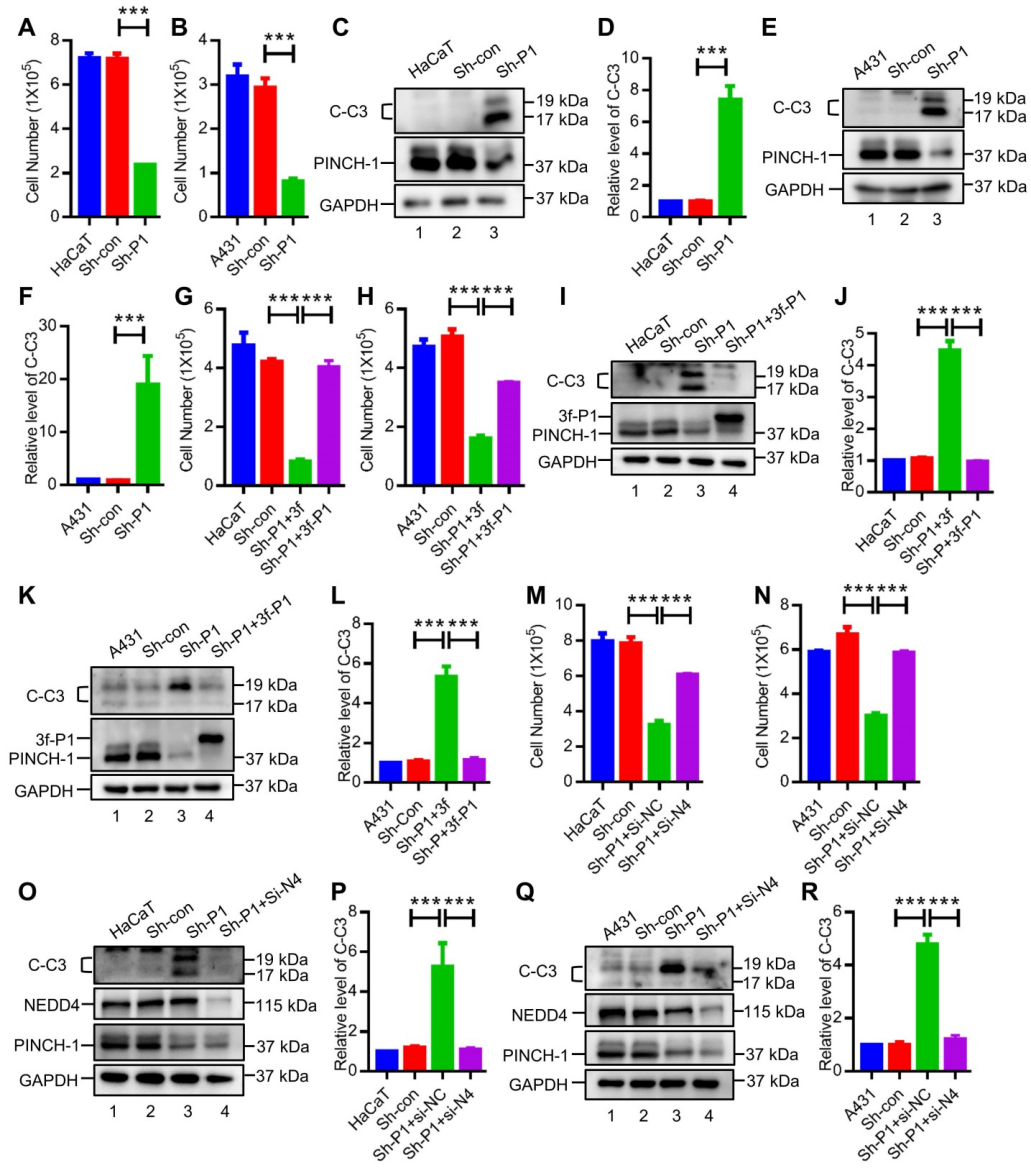
** PINCH-1 regulates cell proliferation and apoptosis through NEDD4. (A-F)** HaCaT **(A and C-D)** and A431** (B and E-F)** cells were infected with Sh-P1 or Sh-con lentivirus and cultured in six-well plates (1 × 10^5^ cells/well) for 3 d. The proliferation of the wild type and infected cells was assessed by counting cell numbers (**A-B**, n = 3). Apoptosis was assessed by WB analysis of cleaved caspase 3 (C-C3). In parallel WB experiments, the samples were probed with anti-PINCH-1 or anti-GAPDH Abs **(C and E)**. The C-C3 levels in the infected HaCaT **(D)** and A431 **(F)** cells were quantified and compared to those in the WT cells (normalized to 1, n = 3). **(G-L)** HaCaT **(G and I-J)** and A431** (H and K-L)** cells were infected with Sh-con or Sh-P1 for 2 d, and the Sh-P1 infectants were then infected with 3f or 3f-PINCH-1 (3f-P1) lentivirus for 3 d. Cell proliferation and apoptosis were assessed by counting cell numbers (**G-H**, n = 3) and C-C3 WB** (I and K)** as described above. The C-C3 levels in the infected HaCaT **(J)** and A431** (L)** cells were quantified and compared to those in the WT cells (normalized to 1, n = 3). **(M-R)** HaCaT **(M and O-P)** and A431** (N and Q-R)** cells were infected with Sh-P1 or Sh-con lentivirus for 2 d and then transfected with Si-NEDD4 (Si-N4) or Si-NC for 3 d as indicated in the figure. Cell proliferation and apoptosis were assessed by counting the cell numbers (**M-N**, n = 3) and C-C3 WB** (O and Q)** as described above. The C-C3 levels in the infected HaCaT** (P)** and A431 **(R)** cells were quantified and compared to those in the WT cells (normalized to 1, n = 3). Data are presented as mean ± SEM using one-way ANOVA with Tukey' post-hoc analysis, ***P < 0.001.

We next sought to determine the functional significance of PINCH-1-mediated regulation of IGF-1R expression. IGF-1R is known to be critical for cell proliferation and survival. Knockdown of PINCH-1, which reduced the level of IGF-1R (Figure 4), markedly inhibited the proliferation of HaCaT (Figure 5A) and A431 (Figure 5B) cells. Furthermore, the results of flow cytometry with Annexin V-FITC and propidium iodide (PI) showed that depletion of PINCH-1 from HaCaT and A431 cells significantly increased apoptosis (Figure S3). PINCH-1 deficiency-induced increase of apoptosis was confirmed by WB analysis of cleaved caspase 3 (Figure 5C-F, lane 3), another marker of apoptosis.

Re-expression of 3f-PINCH-1 in PINCH-1 knockdown HaCaT and A431 cells restored cell proliferation (Figure 5G-H) and survival (Figure 5I-L, lane 4; Figure S3). Importantly, depletion of NEDD4 from PINCH-1 deficient HaCaT and A431 cells reversed to a large extent PINCH-1 deficiency-induced inhibition of cell proliferation (Figure 5M-N) and survival (Figure 5O-R, lane 4). Thus, PINCH-1 promotes cell proliferation and survival through, at least in part, NEDD4. In control experiments, knockdown of NEDD4 from wild type HaCaT cells increased the IGF-1R level (Figure S4A-B). However, neither the level of cleaved caspase 3 (Figure S4C) nor cell proliferation (Figure S4D) were significantly changed in response to NEDD4 knockdown. Thus, while PINCH-1 deficiency-induced down-regulation of IGF-1R expression significantly inhibited cell survival and proliferation (Figure 5), increase of IGF-1R expression above the normal level of IGF-1R in wild type HaCaT cells did not significantly alter cell survival or proliferation under the experimental condition used. Overexpression of IGF-1R in PINCH-1 knockdown HaCaT cells, which expressed significantly reduced level of IGF-1R compared to wild type or control HaCaT cells (Figure S5A, compare lane 3 with lanes 1 and 2), reversed PINCH-1 deficiency-induced defects in cell survival (Figure S5B) and proliferation (Figure S5C). These results confirm that PINCH-1 knockdown-induced defects in cell survival and proliferation are caused by IGF-1R deficiency.

### PINCH-1 regulates IGF-1 receptor expression and proliferation in the epidermis *in vivo*

**Figure 6:**
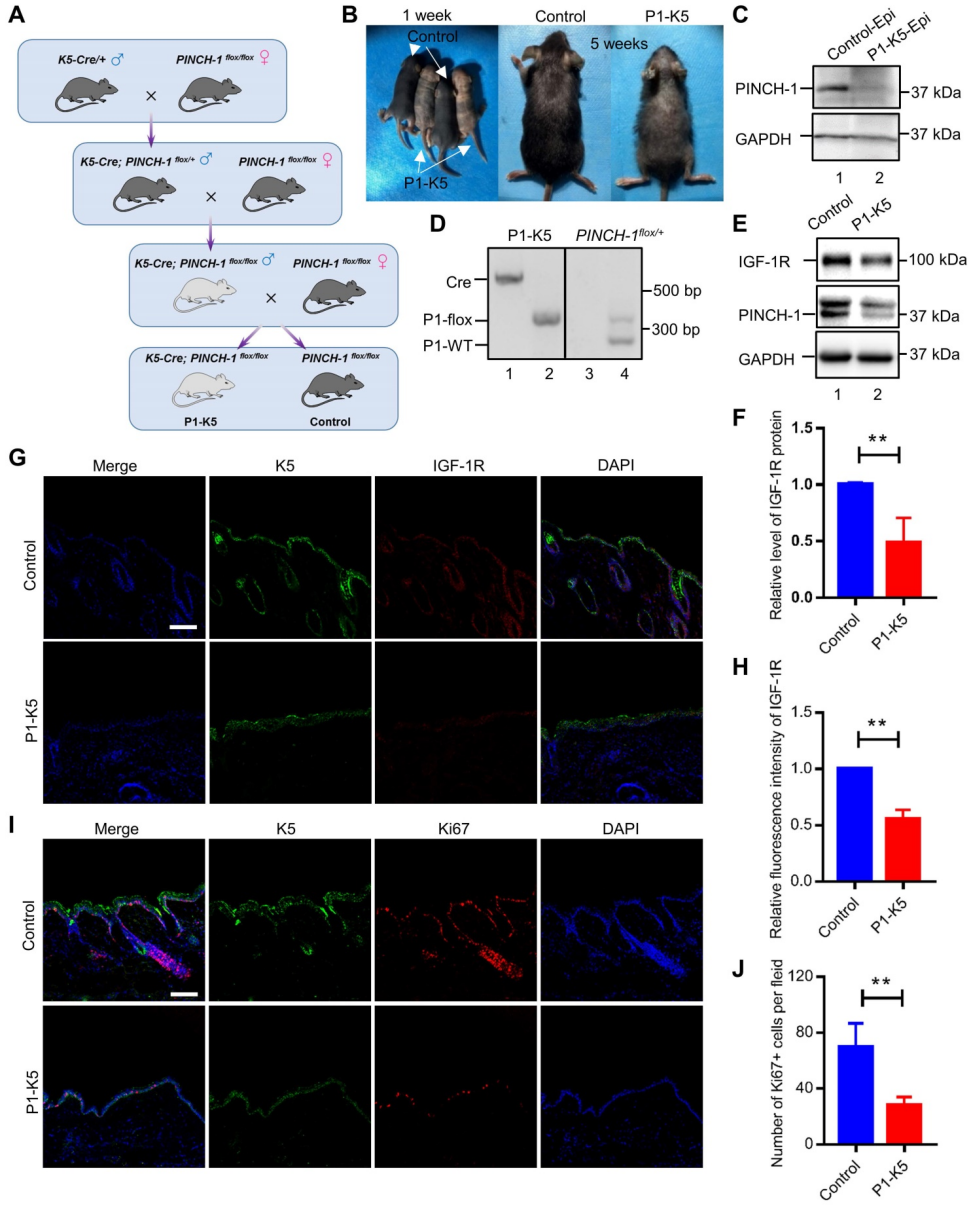
** PINCH-1 regulates IGF-1R expression and cell proliferation in the epidermis *in vivo*. (A)** Mating strategy for generating the P1-K5 and control mice. **(B)** Photographs of the P1-K5 and control mice at 1 and 5 weeks of age.** (C)** The genotypes of the P1-K5 (left) and *PINCH-1 ^flox/+^* mice (right) were analyzed by PCR as described in the Methods. **(D)** Epidermal lysates (Epi) from the control and P1-K5 mice at 65 days of age were analyzed by WB with Abs for PINCH-1 and GAPDH (as a control). **(E)** The lysates of back skin tissues from the control and P1-K5 mice at 5 weeks of age were analyzed by WB with Abs for IGF-1R, PINCH-1 or tubulin (as a control). **(F)** The IGF-1R levels in the skin tissues of P1-K5 mice were quantified and compared to that of the control mice (normalized to 1, n = 3). **(G-J)** The back skin tissues from the control and P1-K5 mice treated with DMBA/TPA for 20 weeks were immunofluorescently stained with DAPI (blue) and Abs for IGF-1R (red) and K5 (green) **(G)** or Abs for Ki67 (red) and K5 (green)** (I)**. Scale bars = 100 μm. **(H)** The immunofluorescence intensities of IGF-1R in the skin tissue sections of the P1-K5 mice were quantified and compared to those in the control mice (normalized to 1, n = 3). **(J)** The number of Ki67 positive cells in the skin tissue sections of P1-K5 mice were quantified and compared to those in the control mice (n = 3). Data are presented as mean ± SEM using t-test analysis, **P < 0.01.

We next investigated the functions of PINCH-1 in regulation of IGF-1R and cell proliferation in the epidermis *in vivo*. To do this, we crossed mice carrying loxP-flanked *PINCH-1* gene (*PINCH-1*^flox/flox^) with mice expressing the Cre recombinase under the control of the K5 promoter 42, 50, 67 using a mating strategy as outlined in Figure 6A. Consistent with previous studies 50, the *K5-Cre; Pinch1^flox/flox^* mice (P1-K5) survived and were relatively healthy. However, compared with the control littermates, the P1-K5 mice were born with relatively sparse hair and uneven deposition of melanin. With time, the hair became sparser and more disheveled, and some areas of the skin were bald (Figure 6B). The genotype of the P1-K5 mice was confirmed by PCR analyses (Figure 6C, lanes 1 and 2)*.* WB analyses showed that PINCH-1 was almost completely eliminated from the epidermal tissues of the P1-K5 mice (Figure 6D, lane 2). WB analyses of the back skin tissues showed that the level of IGF-1R in the skin tissues from the P1-K5 mice was significantly reduced compared to that of the control mice (Figure 6E, compare lanes 1 and 2; Figure 6F). Consistent with the defects in the gross skin phenotype 50, microscopic analysis showed that the skin of P1-K5 mice contained sparse and abnormal hair follicles and hyperthickened interfollicular epidermis (Figure 6G, bottom panels). Immunofluorescence staining analysis showed that the expression of IGF-1R in the skin tissues of the P1-K5 mice was reduced compared with that in the control mice (Figure 6G-H). Furthermore, knockout of PINCH-1 markedly reduced the number of Ki67 positive cells (Figure 6I-J). Thus, consistent with the studies in cultured cells, knockout of PINCH-1 from the epidermis in mice significantly reduced the IGF-1R level and cell proliferation *in vivo*.

### Ablation of PINCH-1 inhibits skin tumor growth in mice

**Figure 7:**
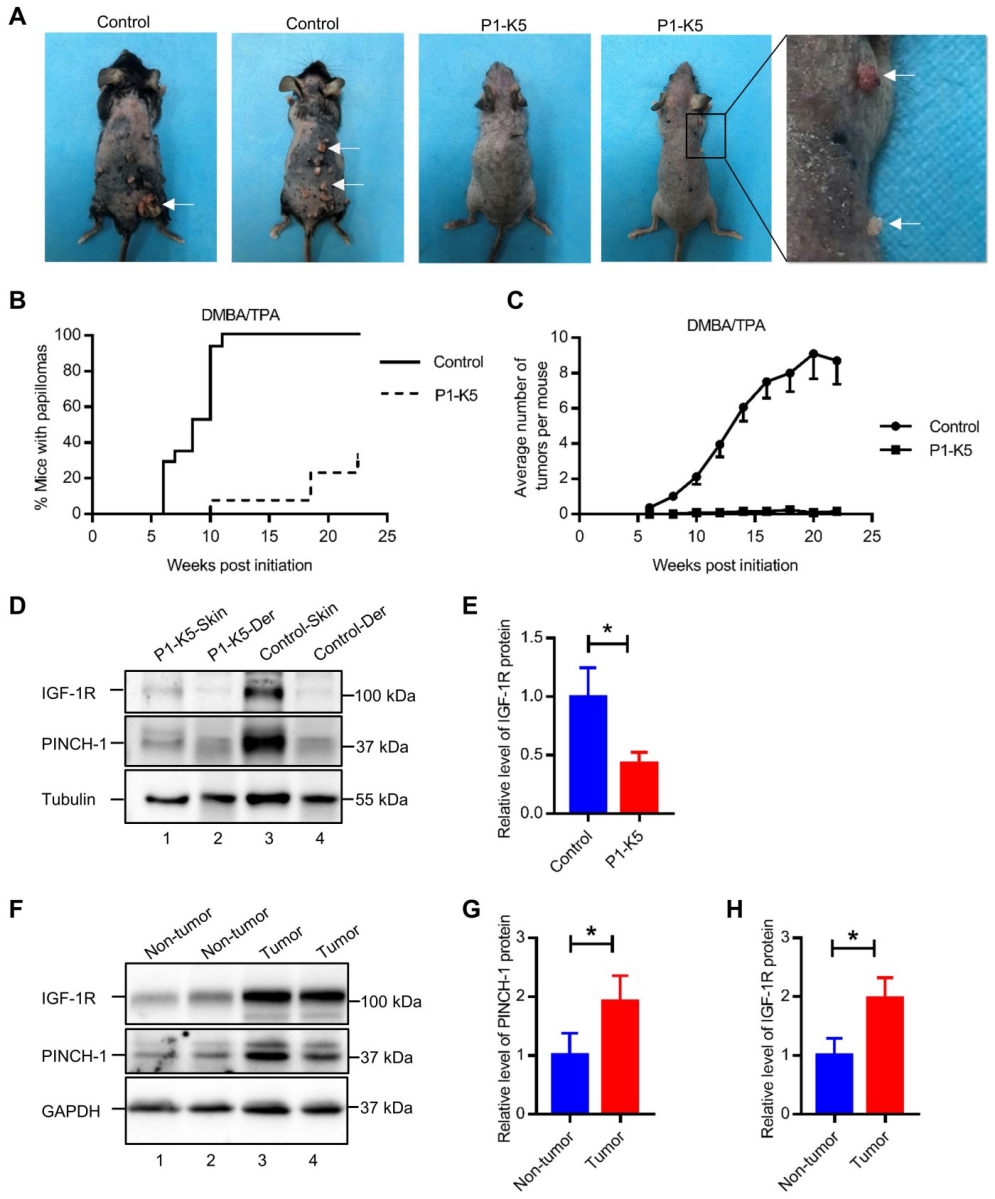
** PINCH-1 promotes skin tumor growth *in vivo*. (A)** Gross appearance of tumors in the DMBA/TPA-treated control and P1-K5 mice at 18 weeks. **(B)** Percentages of the control and P1-K5 mice with papillomas after treatment with DMBA/TPA for 0 to 24 weeks (n≥ 13). **(C)** Average number of tumors per mouse in the DMBA/TPA-treated control and P1-K5 groups (n ≥ 13). **(D)** The lysates of the back skin tissues and the dermis (Der) from the control and P1-K5 mice treated with DMBA/TPA for 20 weeks were analyzed by WB with Abs for IGF-1R, PINCH-1 and tubulin (as a control). **(E)** The IGF-1R levels in the skin tissues of the P1-K5 mice were quantified and compared to those in the control mice (normalized to 1, n = 3). **(F)** The lysates from skin tumor tissues and non-tumor skin tissues of the control mice treated with DMBA/TPA for 20 weeks were analyzed by WB with Abs for IGF-1R, PINCH-1 and GAPDH (as a control). **(G-H)** The PINCH-1 **(G)** IGF-1R** (H)** and levels in the skin tumor tissues were quantified and compared to those in the non-tumor skin tissues (normalized to 1, n = 3). Data are presented as mean ± SEM using t-test analysis, *P < 0.05.

IGF-1R is known to play a pivotal role in promoting tumor growth 9-12. The findings that ablation of PINCH-1 from A431 epidermoid carcinoma cells inhibited IGF-1R expression, cell proliferation and survival prompted us to test whether PINCH-1 plays a role in regulation of skin tumor growth in vivo. To test experimentally the function of PINCH-1 in skin tumor growth in vivo, we treated P1-K5 mice and control littermates with DMBA and TPA following a previously described protocol 63, 68-70. K5 positive cells are mainly located in the basal layer of the skin epidermis to regulate the homeostasis of the epidermis, and they are also critically involved in the development of skin squamous cell carcinoma 57. As expected, papillomas were observed in the control mice beginning at week 6, and by week 10 almost all control mice had developed tumors (Figure 7A-C). By contrast, no papillomas were observed in P1-K5 mice until week 10 and at this time point only 7.7% of the P1-K5 mice developed papillomas (Figure 7B).

**Figure 8:**
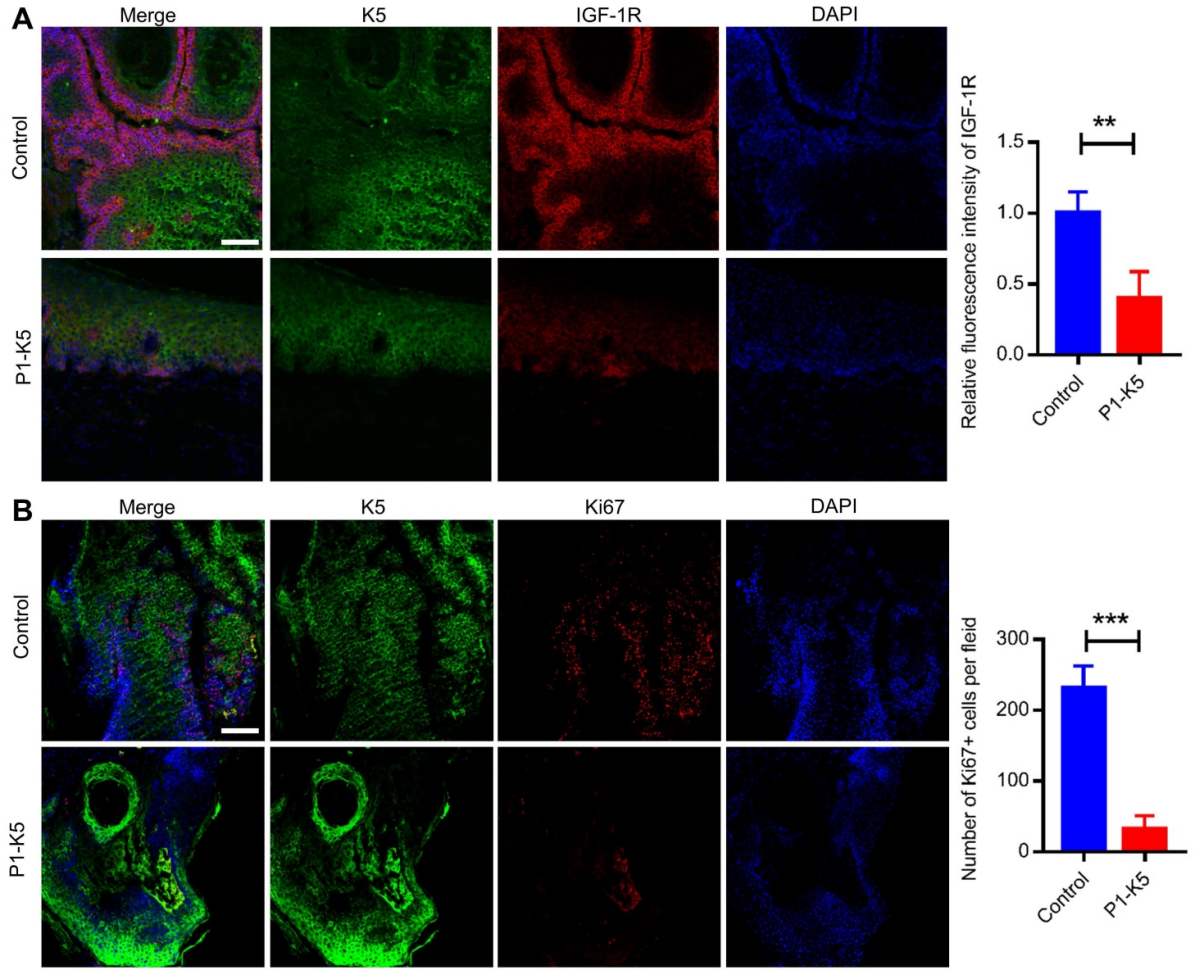
** PINCH-1 promotes IGF-1R expression and cell proliferation in skin tumors. (A-B)** The control and P1-K5 mice were treated with DMBA/TPA for 20 weeks. Tissue sections of the skin tumor from the control and P1-K5 mice were immunofluorescently stained with DAPI (blue) and Abs for IGF-1R (red) and K5 (green) **(A)** or Abs for Ki67 (red) and K5 (green) **(B)**. Scale bars = 100 μm. Right panel in **A**, the immunofluorescence intensities of IGF-1R in the skin tumor tissue sections of the P1-K5 mice were quantified and compared to those in the control mice (normalized to 1, n = 3). Right panel in **B**, the number of Ki67 positive cells in the skin tumor tissue sections of P1-K5 mice were quantified and compared to those in the control mice (n = 3). Data are presented as mean ± SEM using t-test analysis, **P < 0.01, ***P < 0.001.

Furthermore, despite prolonged (up to 24 weeks) exposure to the carcinogens DMBA/TPA, the majority (69.2%) of the P1-K5 mice remained free of skin tumor (Figure 7B). In the relatively small percentage of the P1-K5 mice that developed papillomas, the average number of papillomas per mouse was significantly smaller than that of control mice (Figure 7C). In fact, while the majority of the papillomas in the control mice grew larger over time, many of the papillomas in the P1-K5 mice bearing papillomas failed to grow and were eventually lost. IGF-1R was abundantly expressed in the skin of the DMBA/TPA treated control mice, in which PINCH-1 was also highly expressed (Figure 7D, lane 3), whereas relatively low levels of PINCH-1 and IGF-1R were detected in the dermis of these mice (Figure 7D, lane 4). Ablation of PINCH-1 from the epidermis markedly reduced the level of IGF-1R despite treatment with DMBA/TPA (Figure 7D, compare lane 1 with lane 3; Figure 7E), confirming a critical role of PINCH-1 in regulation of IGF-1R expression. Of note, the level of PINCH-1 in the mouse skin tumors was markedly increased compared to that of the normal control skin (Figure 7F, compare lanes 3 and 4 with lanes 1 and 2; Figure 7G). Concomitantly, the level of IGF-1R was also increased in PINCH-1 rich skin tumor tissues (Figure 7F, compare lanes 3 and 4 with lanes 1 and 2; Figure 7H). Immunofluorescence staining experiments confirmed that the IGF-1R level in the epidermis was reduced in response to loss of PINCH-1 despite the presence of DMBA/TPA (Figure S6A). As expected, the IGF-1R level was markedly reduced in the skin tumors of P1-K5 mice (Figure 8A, bottom panels) compared to the skin tumors of control mice (Figure 8A, top panels). Finally, concomitant to the reduction of the IGF-1R level, cell proliferation in the epidermis was significantly reduced in response to ablation of PINCH-1 despite the presence of DMBA/TPA (Figure S6B). Cell proliferation in the skin tumors of P1-K5 mice (Figure 8B, bottom panels) was also markedly reduced compared to that in the skin tumors of control mice (Figure 8B, top panels). Collectively, these results suggest that depletion of PINCH-1 effectively inhibits IGF-1R expression, cell proliferation and skin tumor growth *in vivo*.

## Discussion

The level of IGF-1R is frequently increased in malignant tumors, which has been well recognized as a key event in promoting cancer cell proliferation, survival and tumor growth 11. The molecular mechanisms by which the IGF-1R level is regulated, however, are incompletely understood. In the current study, we have identified PINCH-1 as a positive regulator of IGF-1R expression in A431 squamous carcinoma cells in culture as well as skin tumors in mice. Consistent with a critical role of IGF-1R in promoting cancer cell proliferation, survival and tumor growth, depletion of PINCH-1 was sufficient to inhibit cancer cell proliferation, survival and tumor growth.

How does PINCH-1 promote IGF-1R expression? We have found that PINCH-1 binds directly to NEDD4. Furthermore, we have mapped a major PINCH-1-binding site to the NEDD4 C2 domain, which is known to mediate the interaction with GRB10 29. Depletion of PINCH-1 in cells increased whereas overexpression of PINCH-1 in cells reduced the formation of the GRB10/NEDD4 complex. These findings, together with the fact that the GRB10/NEDD4 complex is known to promote cellular degradation of IGF-1R 29, suggest that PINCH-1 promotes the cellular level of IGF-1R through, at least in part, inhibition of the GRB10-NEDD4 complex formation. Consistent with this, depletion of NEDD4 effectively blocked PINCH-1 deficiency-induced down-regulation of IGF-1R expression (Figure 4G-H).

While our findings provide strong evidence supporting a crucial role of the PINCH-1-NEDD4-IGF-1R signaling axis in regulation of skin cancer cell proliferation, survival and tumor growth, they do not rule out the possibility that PINCH-1 may also participate in other signaling pathways that are pertinent to regulation of cancer cell proliferation and survival. Indeed, previous studies by us and others have shown that PINCH-1 can interact with multiple signaling proteins including ILK 37, Nck-2 60, RSU-1 61, 62, EPLIN 50, myoferlin 53 and Notch-2 55, which may also contribute to the regulation of cancer cell proliferation and survival. Nevertheless, the fact that depletion of NEDD4 from PINCH-1 deficient squamous carcinoma cells restored to a large extent cell proliferation and survival (Figure 5M-R) strongly argues that the PINCH-1-NEDD4-IGF-1R signaling axis delineated in the current study represents one of the major, if not the only, signaling pathways through which PINCH-1 regulates squamous carcinoma cell proliferation and survival.

PINCH-1 as well as its downstream effector IGF-1R are expressed in not only skin tumors but also normal epidermis, albeit their expression levels in the latter are considerably lower than those in the tumors (Figure 7F-G). Depletion of PINCH-1 from HaCaT keratinocytes in culture and normal epidermis in mice, like that from A431 squamous carcinoma cells and chemical carcinogen-induced skin tumors in mice, also reduced the IGF-1R level. Thus, the PINCH-1-NEDD4-IGF-1R signaling axis delineated in the current study likely also operates in normal epidermis. This may explain, at least in part, the skin and hair defects of the PI-K5 mice observed by us and others (Figure 6B) 50.

The incidence of skin cancer has been increasing continuously, which has become a major public health problem in the world 4, 48, 49. Development of effective therapeutic approaches to mitigate the progression of skin cancer, therefore, is of great clinical significance. Of note, while ablation of PINCH-1 from the K5 positive cells effectively suppresses the genesis and progression of skin cancer (Figure 7A), the P1-K5 mice were relatively healthy. Thus, targeting the PINCH-1-NEDD4-IGF-1R signaling axis in the epidermis may provide an attractive strategy for therapeutic control of the genesis and progression of skin cancer. Finally, it is worth noting that both PINCH-1 and IGF-1R are overexpressed in several other types of cancers including non-small cell lung cancer, colorectal cancer and prostate cancer 54, 58, 59, 71-74. Thus, it will be interesting to test whether the PINCH-1-NEDD4-IGF-1R signaling axis delineated in the current study also contributes to the increase of IGF-1R expression in other cancer types in which both PINCH-1 and IGF-1R are upregulated, and if so, whether inhibition of the PINCH-1-NEDD4-IGF-1R signaling axis can alleviate the progression of these cancers.

## Supplementary Material

Supplementary figures and tables.

## Abbreviations

IGF-1R: insulin like growth factor 1 receptor
NEDD4: Neuronal precursor cell-expressed developmentally downregulated 4
ECM: cell-extracellular matrix
DMBA: 7, 12-dimethylbenz[a]anthracene
TPA: O-tetradecanoylphorbol-13-acetate
K5: keratin 5
BCC: basal cell carcinoma
SCC: squamous cell carcinoma
GRB10: growth factor receptor bound protein 10
PINCH-1: particularly interesting new cysteine-histidine rich protein 1
LIMS1: Lims and senescent cell antigen-like domain 1
ILK: integrin-linked kinase
BMPR2: bone morphogenetic protein receptor type II
*PINCH-1^flox/flox^*: mice carrying loxP-flanked *PINCH-1* gene
