# Supplementary material for: PINCH-1 promotes IGF-1 receptor expression and skin cancer progression through inhibition of the GRB10-NEDD4 complex
Source: Theranostics. 2022 Feb 28;12(6):2613–30. doi: 10.7150/thno.70744 (PMC8965486; doi:10.7150/thno.70744)
Supplement: Supplementary file 1 — Supplementary figures and tables. [file thnov12p2613s1.pdf]

Supplementary Information for

PINCH-1 promotes IGF-1 receptor expression and skin cancer progression through inhibition of the GRB10-NEDD4 complex

Xiaoxiao Wang[1#], Rong Wang[1#], Kun Jiang[1], Maohua Zhu[1], Ling Guo[1*], Chuanyue Wu[2*]

[1]Guangdong Provincial Key Laboratory of Cell Microenvironment and Disease Research, Shenzhen Key Laboratory of Cell Microenvironment, Department of Biology, and Academy for Advanced Interdisciplinary Studies, Southern University of Science and Technology, China

[2]Department of Pathology, University of Pittsburgh School of Medicine, Pittsburgh, PA 15261, USA

[#]These authors contributed equally to this work.

[*]Correspondence: carywu@pitt.edu; guol@sustech.edu.cn

Supplementary Figure 1

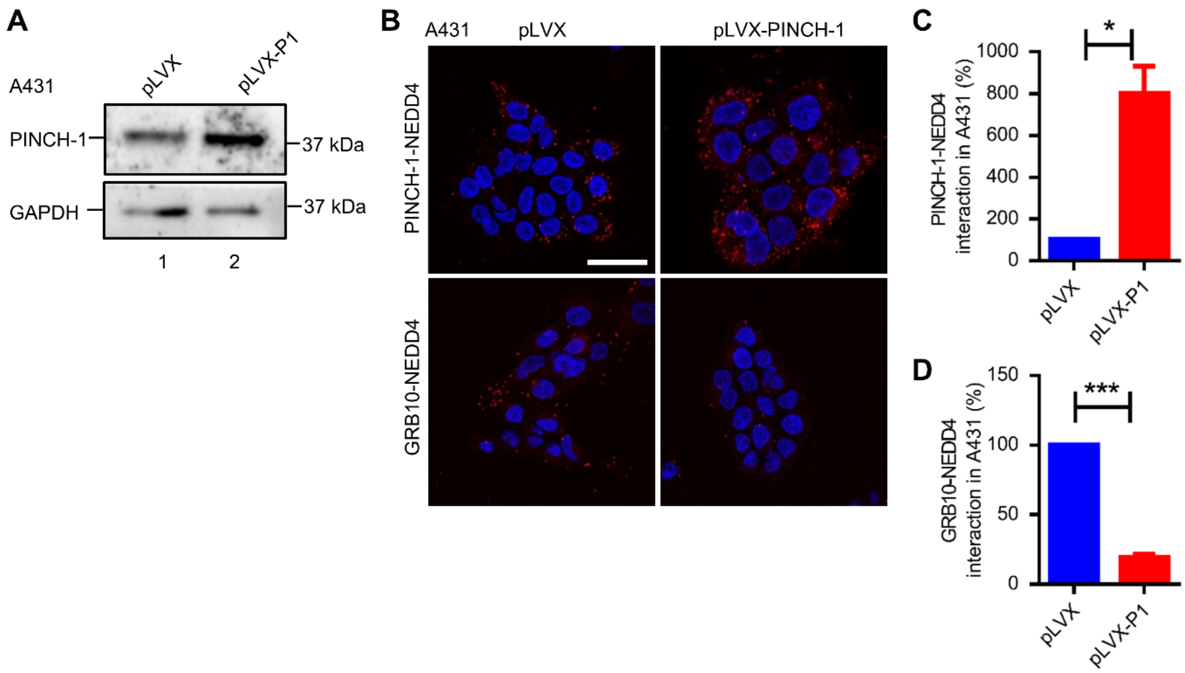

Figure S1. Overexpression of PINCH-1 inhibits the GRB10-NEDD4 complex formation. **(A-D)** A431 cells were infected with lentiviral vectors encoding pLVX or pLVX-PINCH-1 (pLVX-P1) and cultured in medium for 5 d. The cells were analyzed by WB with Abs for PINCH-1 and GAPDH **(A)** or by PLA with Abs for PINCH-1 and NEDD4 or GRB10 and NEDD4 **(B)** as indicated in the figures. The PLA signals (red) and DAPI staining (blue) were visualized under fluorescent microscopy **(B)**. Scale = 50 μm. The PINCH-1-NEDD4 **(C)** and GRB10-NEDD4 **(D)** protein complexes detected by PLA were quantified as described in the Materials and Methods. Data are presented as mean ± SEM using t-test analysis, $^*P < 0.05$, $^{***}P < 0.001$.

Supplementary Figure 2

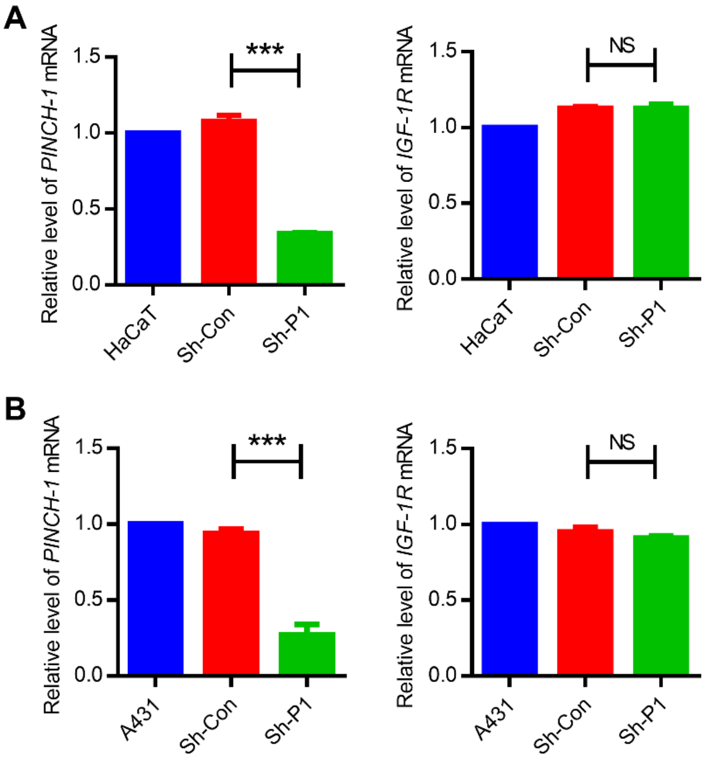

Figure S2. Depletion of PINCH-1 does not reduce the *IGF-1R* mRNA level. (A-B) The mRNA levels of *PINCH-1* and *IGF-1R* in the Sh-P1 or Sh-con lentiviral infected HaCaT **(A)** and A431 **(B)** cells were analyzed by RT-PCR and compared to those of the wild type cells (normalized to 1, n = 3). Data represent mean ±SEM. Statistical significance was calculated using one-way ANOVA with Tukey' post-hoc analysis, ***P < 0.001.

Supplementary Figure 3

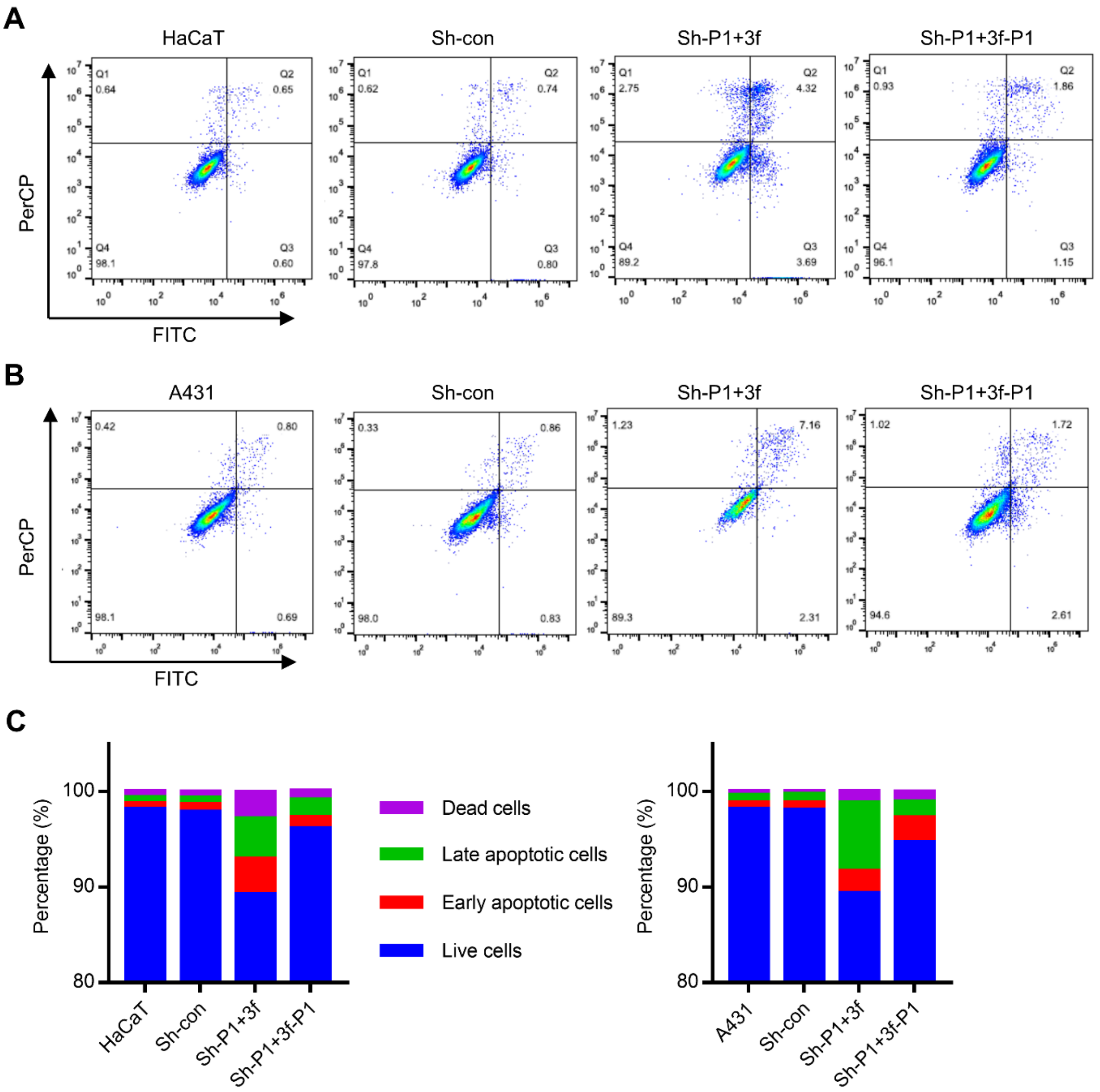

Figure S3. Depletion of PINCH-1 increases apoptosis. (A-C) HaCaT **(A)** and A431**(B)** cells were infected with Sh-con or Sh-P1 for 2 d, and the Sh-P1 infectants were then infected with 3f or 3f-P1 lentivirus. Three days later, apoptosis was analyzed using the Annexin V-FITC/PI Apoptosis Detection Kit and flow cytometric analysis as described in the Materials and Methods.

(C) The percentages of dead cells, late apoptotic cells, early apoptotic cells and live cells in HaCaT **(A)** and A431 **(B)** were quantified.

Supplementary Figure 4

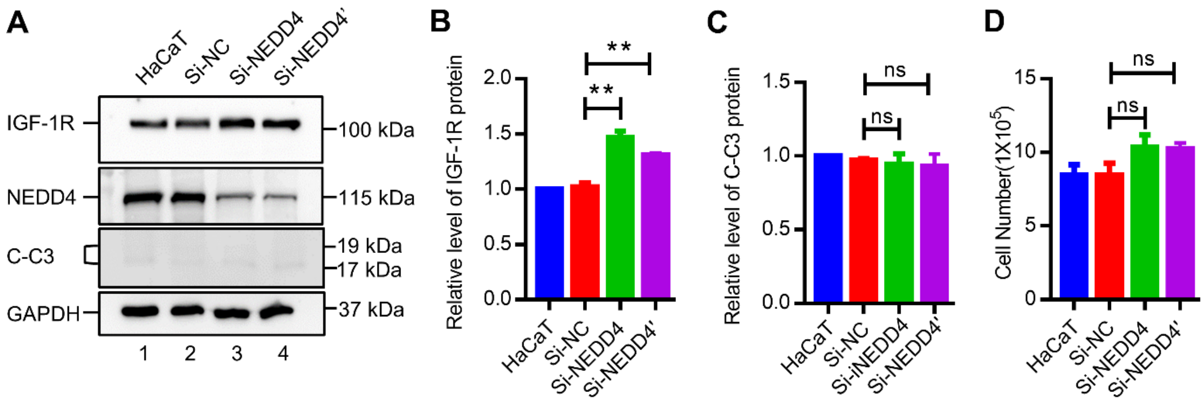

Figure S4. Depletion of NEDD4 from wild type HaCaT cells increases IGF-1R expression. **(A-D)** HaCaT cells were transfected with Si-NEDD4, Si-NEDD4' or Si-NC for 2 d. The cells were analyzed by WB with Abs as indicated **(A)**. The IGF-1R **(B)** and cleaved caspase 3 (C-C3) **(C)** levels in the NEDD4 knockdown cells were quantified and compared to those in the wild-type HaCaT cells (normalized to 1, n = 3). **(D)** The proliferation of the wild type and infected HaCaT cells was assessed by counting cell numbers as described in the Methods (n = 3). Data are presented as mean ± SEM using one-way ANOVA with Tukey' post-hoc analysis, **P < 0.01.

Supplementary Figure 5

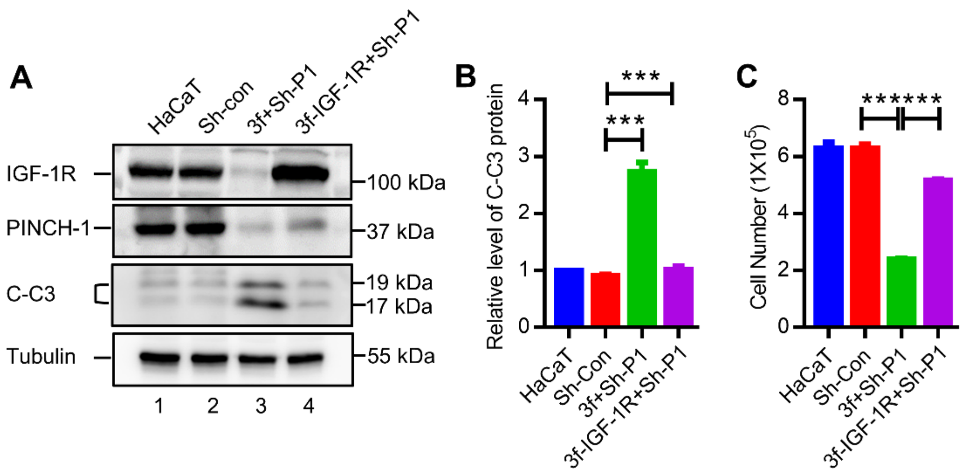

Figure S5. Overexpression of IGF-1R reverses the PINCH-1 deficiency-induced defects in cell survival and proliferation. (A-C) HaCaT cells were infected with Sh-con or Sh-P1 for 2 d, and the Sh-P1 infectants were then infected with 3f or 3f-IGF-1R lentivirus. Three days later, the infectants and wild type HaCaT cells were analyzed by WB with Abs as indicated **(A)**. **(B)** The cleaved caspase 3 (C-C3) levels in the infectants were quantified and compared to those in the wild type HaCaT cells (normalized to 1, n = 3). **(C)** The proliferation of the wild type and infected HaCaT cells was assessed by counting cell numbers as described in the Methods (n = 3). Data are presented as mean ± SEM using one-way ANOVA with Tukey' post-hoc analysis, ***P < 0.001.

Supplementary Figure 6

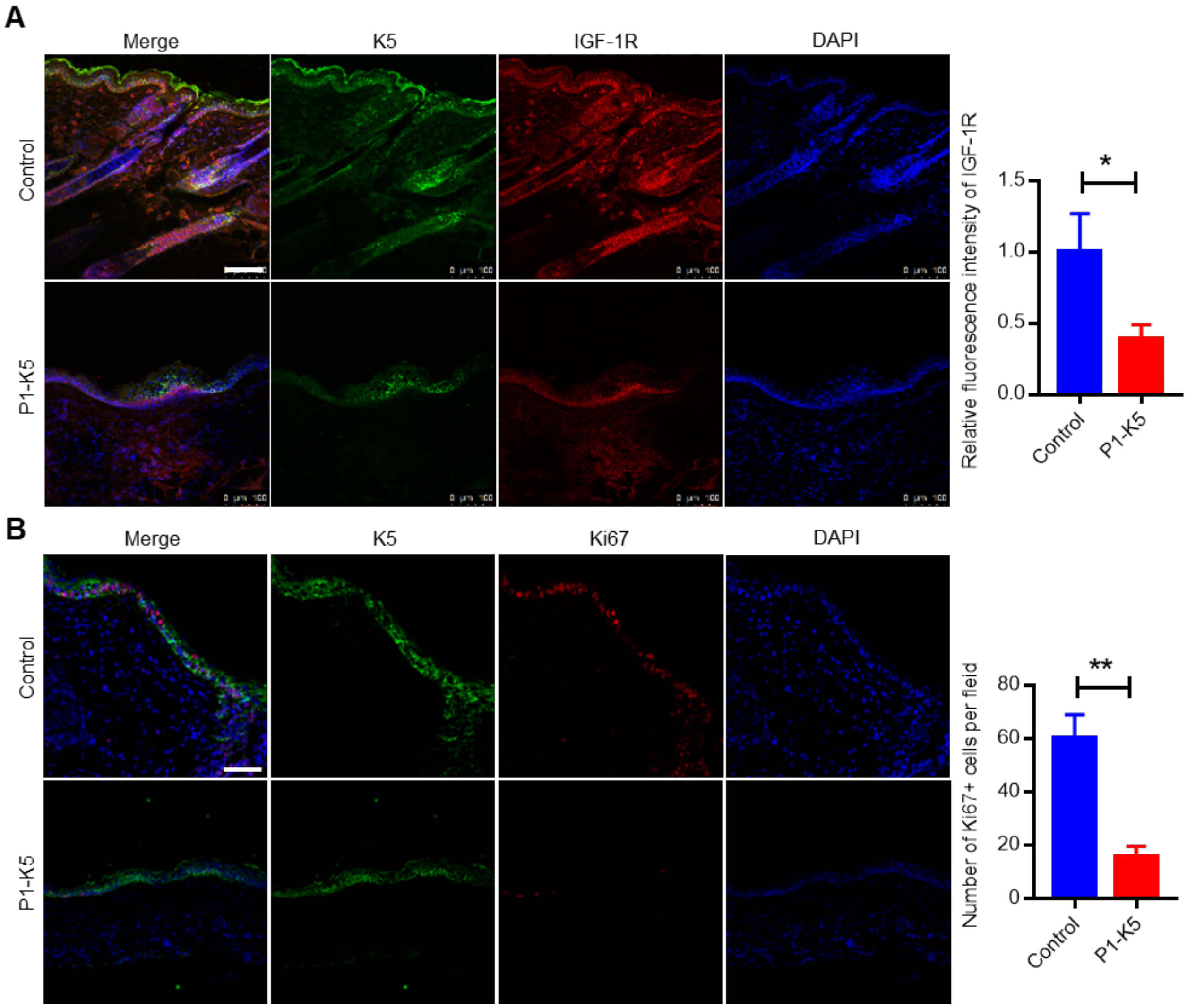

Figure S6. PINCH-1 regulates IGF-1R expression and cell proliferation in the DMBA/TPA-treated mouse epidermis. (A-B) The control and P1-K5 mice were treated with DMBA/TPA for 20 weeks. Skin tissue sections of the control and P1-K5 mice were immunofluorescently stained with DAPI (blue) and Abs for IGF-1R (red) and K5 (green) **(A)** or Abs for Ki67 (red) and K5 (green) **(B)**. Scale bars = 100 μm. Right panel in **A**, the immunofluorescence intensities of IGF-1R in the skin tissue sections of the P1-K5 mice were quantified and compared to those in the control mice (normalized to 1, n = 3). Right panel in **B**,

the number of Ki67 positive cells in the skin tissue sections of P1-K5 mice were quantified and compared to those in the control mice (n = 3). Data are presented as mean ± SEM using t-test analysis, *P < 0.05, **P < 0.01.

Supplementary Table 1

Table S1: List of PCR primers for mouse genotyping

Gene	Sequence (5' to 3')	
Cre	Sense	GATCTCCGGTATTGAAACTCCAGC
	Antisense	GCTAAACATGCTTCATCGTCGG
PINCH-1 (fl/fl)	Sense	CCCAGAAGGACTCTTTTATGAG
	Antisense	CTTGGAGAAGAAGTACTCAGG

Supplementary Table 2

Table S2: List of primers for QRT-PCR

Gene	Sequence (5' to 3')	
GAPDH (h)	Sense	CCAGAACATCATCCCTGCCTCTACT
	Antisense	GGTTTTTCTAGACGGCAGGTCAGGT
PINCH-1 (h)	Sense	CCGCTGAGAAGATCGTGAAC
	Antisense	GGGCAAAGAGCATCTGAAAG
IGF-1R (h)	Sense	GGCATACCTCAACGCCAATA
	Antisense	CAGCCCTTTCCCTCCTTT
